# Lipids mediate supramolecular outer membrane protein assembly in bacteria

**DOI:** 10.1126/sciadv.adc9566

**Published:** 2022-11-02

**Authors:** Melissa N. Webby, Abraham O. Oluwole, Conrado Pedebos, Patrick G. Inns, Anna Olerinyova, Dheeraj Prakaash, Nicholas G. Housden, Georgina Benn, Dawei Sun, Bart W. Hoogenboom, Philipp Kukura, Shabaz Mohammed, Carol V. Robinson, Syma Khalid, Colin Kleanthous

**Affiliations:** ^1^ Department of Biochemistry, South Parks Road, University of Oxford, Oxford OX1 3QU, UK.; ^2^ Physical and Theoretical Chemistry Laboratory, Department of Chemistry, University of Oxford, Oxford OX1 3QZ, UK.; ^3^ The Kavli Institute for Nanoscience Discovery, South Parks Road, Oxford OX1 3QZ, UK.; ^4^ London Centre for Nanotechnology, University College London, London WC1H 0AH, UK.; ^5^ Institute of Structural and Molecular Biology, University College London, London WC1E 6BT, UK.; ^6^ Structural Biology, Genentech Inc., South San Francisco, USA.; ^7^ Department of Physics and Astronomy, University College London, WC1E 6BT London, UK.; ^8^ Department of Chemistry, Chemistry Research Laboratory, University of Oxford, Oxford OX1 3QZ, UK.; ^9^ Mechanistic Proteomics, Rosalind Franklin Institute, Harwell Campus, Didcot OX11 OFA, UK.

## Abstract

β Barrel outer membrane proteins (OMPs) cluster into supramolecular assemblies that give function to the outer membrane (OM) of Gram-negative bacteria. How such assemblies form is unknown. Here, through photoactivatable cross-linking into the *Escherichia coli* OM, coupled with simulations, and biochemical and biophysical analysis, we uncover the basis for OMP clustering in vivo. OMPs are typically surrounded by an annular shell of asymmetric lipids that mediate higher-order complexes with neighboring OMPs. OMP assemblies center on the abundant porins OmpF and OmpC, against which low-abundance monomeric β barrels, such as TonB-dependent transporters, are packed. Our study reveals OMP-lipid-OMP complexes to be the basic unit of supramolecular OMP assembly that, by extending across the entire cell surface, couples the requisite multifunctionality of the OM to its stability and impermeability.

## Teaser

Lipids tether functionally-diverse proteins together creating complexes that maintain stability of the bacterial outer membrane.

## INTRODUCTION

The outer membrane (OM) is a important barrier to the entry of several classes of antibiotics in Gram-negative bacteria, a property ascribed to its asymmetric distribution of outer leaflet lipopolysaccharides (LPSs) and inner leaflet phospholipids (PLs) (*1*–*4*). OM integrity is necessarily compromised by numerous outer membrane proteins (OMPs)—~1.5% of the *Escherichia coli* (*E. coli*) chromosome encodes OMPs (*5*)—that mediate metabolite exchange (*3*), nutrient import (*6*), OM stabilization (*7*), hydrolysis of antimicrobial peptides (*8*), ejection of antibiotics (*9*), and adherence to surfaces (*10*, *11*). Most OMPs are β barrels of between 8 and 36 β strands. OMPs and LPS are incorporated into the membrane by essential β barrel proteins, BamA and LptD. BamA (and lipoprotein partners BamBCDE) catalyze OMP insertion (*12*–*14*), while LptD (and lipoprotein partner LptE) inserts LPS (*15*–*17*).

The standard model of the OM is that of a gel-like or liquid crystalline layer in which OMPs are randomly distributed (*18*–*26*). However, recent live-cell imaging suggests that the *E. coli* OM has a high degree of organization, which is at odds with this classical view: (i) OMPs form punctate clusters or islands that exhibit spatiotemporal behavior in which old OMPs segregate to the poles during growth, and new OMPs are inserted predominantly at division sites (*27*–*29*). (ii) Atomic force microscopy (AFM) shows the OM to have distinct LPS- and OMP-rich regions and that OMP-rich regions dominate this phase-separated landscape (*30*).

How OMPs are accommodated in an organized OM is unknown. To address this question, we devised a photoactivatable cross-linking strategy to capture OMP near-neighbors in live *E. coli* cells. We defined the cross-linked species, reconstituted higher-order complexes in vitro, and incorporated the association principles stemming from these data into simulations. We show that the asymmetric lipids that render the OM an effective impermeability barrier also mediate promiscuous interactions between OMPs, acting as noncovalent adhesive to stabilize OMP networks across the bacterial surface.

## RESULTS

### Strategy for capturing OMP near-neighbor contacts in live *E. coli* MG1655 cells

OMPs exist predominantly as monomers or trimers in Gram-negative bacteria. We set out to define near-neighbor contacts for each class, focusing on trimeric OmpF and monomeric Ferrienterobactin receptor (FepA) and Outer membrane cobalamin transporter (BtuB). OmpF monomers are 16-stranded β barrels that form nonspecific pores through which small molecules diffuse (*M*_w_ < 600 Da), including antibiotics (*31*). OmpF and its structural homolog OmpC are some of the most abundant OMPs in *E. coli*, accounting for more than 40% of the OM proteome (*32*). FepA and BtuB are TonB-dependent transporters (TBDTs) in which a plug domain occludes the central channel of the 22-stranded β barrel (*6*). FepA and BtuB are less abundant than OmpF, but through their energized transport of scarce nutrients across the OM, the siderophore ferric-enterobactin and vitamin B_12_ respectively fulfill equally important roles.

We first established the relative cellular distribution of our target OMPs using fluorescence imaging in *E. coli* MG1655 cells. OmpF, FepA, and BtuB were specifically labeled with fluorescently tagged, high-affinity colicins inactivated for toxicity (*27*, *33*–*35*) and visualized by total internal reflection fluorescence microscopy (fig. S1, A and B). BtuB and FepA were organized into OMP islands, as described previously (*27*), which contained ~20 to 30 copies of each OMP as indicated by photobleaching analysis (fig. S1E). FepA and BtuB resided within regions that also contained OmpF (fig. S1, A to D), the widespread distribution of which is consistent with recent AFM data (*30*).

**Fig. 1.**
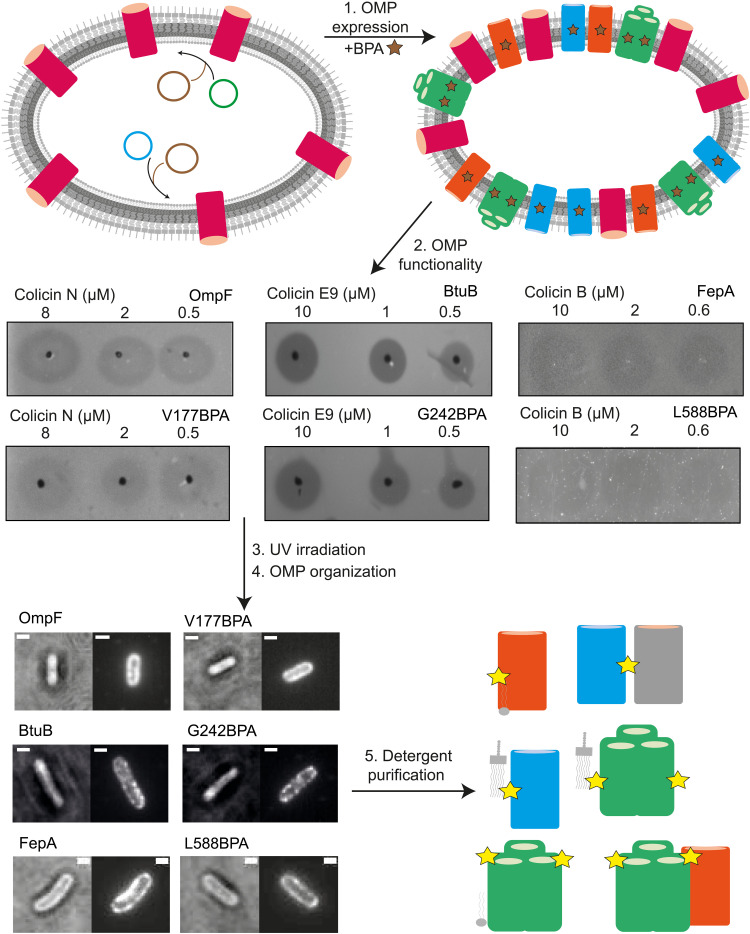
Strategy for identifying OMP near-neighbors by BPA-mediated cross-linking into the *E. coli* OM. Schematic outlining the key steps of the protocol for UV-activated OMP^BPA^ cross-linking, including OmpF (green), BtuB (blue), and FepA (orange). (1) A plasmid (blue and green) encoding the *omp* gene of interest with an amber stop codon (TAG) at a single site was transformed into a knockout cell line devoid of the *omp* of interest, in conjunction with a plasmid for expressing the tRNase (pEVOL-pBpF, brown) required for BPA incorporation. Cells were then grown in the presence of BPA (brown star; shown as a yellow star following UV activation) and *omp* gene expression induced with 0.15% arabinose. (2) A colicin-based cytotoxic assay was used to test for OMP functionality in the OM. The colicins used in the study each require the OMP of interest as a receptor before import and cell killing. Hence, colicin cytotoxicity is a simple readout of appropriate expression/insertion into the OM. Examples of successful killing of *E. coli* cells expressing BPA-incorporated OMPs are shown for each protein target. (3) Cells were exposed to UV light (λ_365_) for 90 min to activate BPA cross-linking. (4) The cellular distribution of the OMP of interest was subsequently reanalyzed, to ascertain whether it was similar to that of the wild-type protein, using fluorescent colicin labels as in figs. S3 to S5. Scale bar, 1 μm. (5) Cells were lysed, the OM was extracted, and OMPs were solubilized with detergent (1% β-OG) and purified by chromatography for further characterization (see Materials and Methods for details).

We next established a photoactivatable cross-linking protocol by which close (<4 Å) associations could be probed (Fig. 1). Para-benzoylphenylalanine (BPA), which cross-links to C─H bonds when exposed to ultraviolet (UV) light, was incorporated into multiple outward-facing, transmembrane positions of the β barrels. Expression of individual *omp* genes, usually containing a single BPA insertion site, was induced from a plasmid in an *E. coli* strain lacking the gene encoding the OMP of interest and grown in the presence of BPA. Functionality was scored by bacteriocin-mediated killing of *E. coli* strains expressing them: colicin N for OmpF, colicin E9 for BtuB, and colicin B for FepA (Fig. 1). Of the 35 BPA sites introduced across the three OMPs, 6 were nonfunctional in colicin toxicity assays: 5 of 19 for OmpF (all located close to subunit interfaces), 1 of 8 for BtuB, and 0 of 8 for FepA (table S1). We interpreted lack of colicin-mediated killing as evidence of inappropriate OMP folding/insertion into the OM and was not investigated further. BPA mutants that supported colicin-mediated killing (figs. S2 to S5 and table S1) were exposed to UV light (λ_365_, ~90 min), after which cell viability was determined by colony-forming units, and the distribution of each OMP was reevaluated by fluorescence microscopy to confirm functionality (Fig. 1). Both viability and OMP patterning were largely unaffected by UV exposure (figs. S3 to S5). The OMs of all UV-treated strains were then disrupted, and each OMP^BPA^ mutant was put through a pipeline of detergent solubilization [1% *n*-octyl-β-d-glucopyranoside (β-OG)] followed by purification (see the Supplementary Materials for details) and biochemical and biophysical characterization to determine the outcomes of cross-linking.

### OMPs are predominantly enveloped by lipids that reflect the asymmetry of the OM

Coarse-grain simulations suggest that direct protein-protein interactions between β barrels are the basis for clustering of heterologous OMPs in the *E. coli* OM (*27*, *36*–*38*). Yet, in only one case was a direct cross-link between two OMPs detected by liquid chromatography–tandem mass spectrometry (LC-MS/MS), involving BtuB W164^BPA^ and BamA Y531 (fig. S6). The same cross-link was identified in two independently prepared samples, suggesting a specific albeit promiscuous BtuB-BamA contact.

**Fig. 2.**
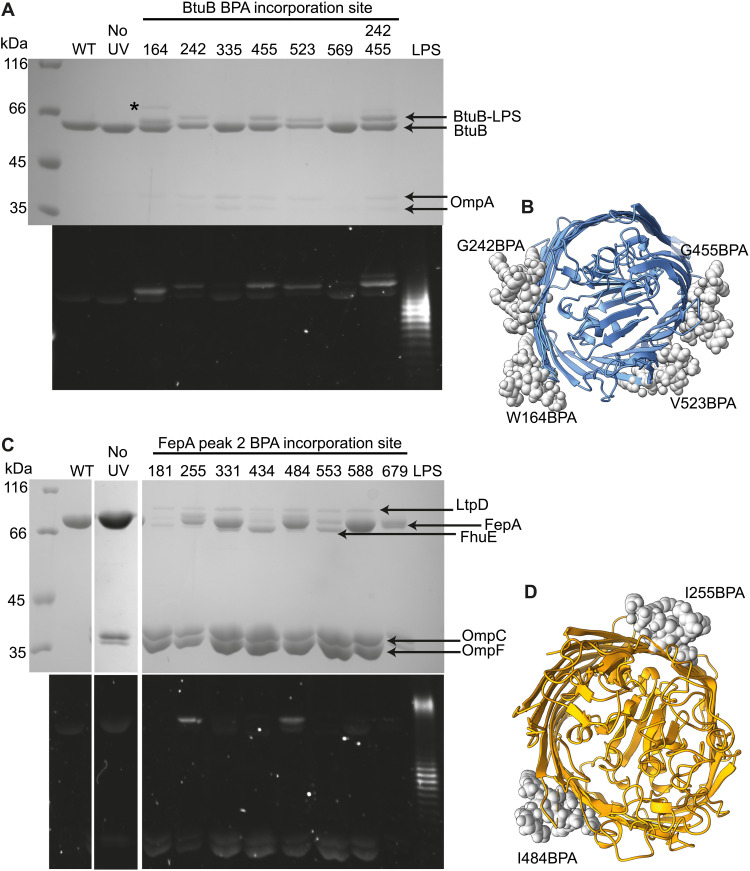
BtuB^BPA^ and FepA^BPA^ cross-linking into the OM enhances associations with other OMPs and lipids. (**A**) Denaturing SDS-polyacrylamide gel of BtuB^BPA^ variants extracted from the OM of *E. coli* RK5016 cells following UV exposure and stained with Coomassie blue for protein (top) and emerald green for LPS (bottom). Several variants cross-link to LPS, as indicated by a gel shift and fluorescence with LPS stain, which is not observed in wild-type BtuB or a no-UV BtuB W164^BPA^ control. Small amounts of OmpA are observed in BtuB variants that contain BPA. The noncovalent recruitment of BamA to BtuB W164^BPA^ (black asterisk) was confirmed by peptide fingerprinting and Western blot. (**B**) Top view of BtuB β barrel showing BPA incorporation sites (gray spheres) where BPA cross-linking into the membrane results in covalent attachment of LPS. (**C**) Denaturing SDS-polyacrylamide gel of FepA^BPA^ variants extracted from the OM of *E. coli* BW25113 Δ*FepA* cells following UV exposure and stained with Coomassie blue for protein (top) and emerald green for LPS (bottom). FepA^BPA^ mutants that have been exposed to UV copurify with the same complement of additional OMPs, OmpF/C, FhuE, and LptD, but their yields vary between BPA incorporation sites. In the absence of UV exposure, there is a notable reduction in the copurification of OMPs. Although the number of LPS cross-links appear minimal (only two are detected on the gel), it is likely that the increased copurification of OmpF/C for FepA^BPA^ mutants arises from lipid-mediated interactions with cross-linked PL and/or with LPS associated noncovalently with porins. (**D**) Top view of FepA β barrel showing BPA incorporation sites (gray spheres) where cross-linking into the membrane results in covalent attachment of LPS.

**Fig. 3.**
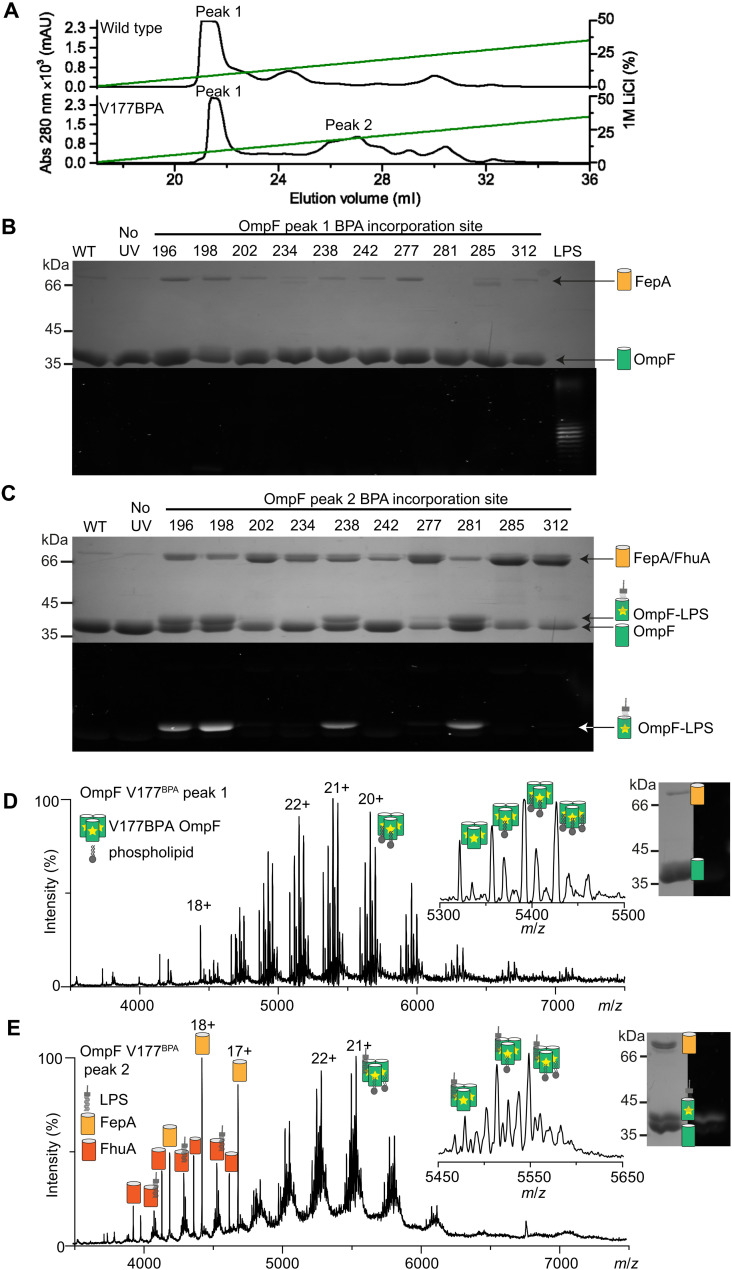
OmpF^BPA^ cross-linking to the asymmetric lipids of the OM stabilizes promiscuous associations with TBDTs. (**A**) Representative UV_280_ absorbance recording from the final anion exchange purification step showing wild-type OmpF eluting in a major peak (peak 1) with a trailing edge. Peak 1 persists following exposure of OmpF^BPA^ mutants to UV and a second peak (peak 2) appears eluting later in the salt gradient; representative profile of OmpF V177^BPA^ is shown. (**B**) OmpF^BPA^ peak 1 samples were analyzed by denaturing SDS-PAGE and stained with Coomassie blue for protein (top) and ProQ emerald green for LPS (bottom). In some cases, exposure of OmpF^BPA^ mutants to UV results in elevated levels of copurified FepA. (**C**) OmpF^BPA^ peak 2 samples were analyzed by denaturing SDS-PAGE and stained with Coomassie blue for protein (top) and ProQ emerald green for LPS (bottom). Cross-links between OmpF^BPA^ mutants and LPS, as indicated by fluorescence following emerald green staining of SDS-PAGE gels, result in enhanced copurification of the TBDTs FepA and FhuA. (**D**) Native-MS spectrum of UV-activated OmpF V177^BPA^ peak 1 shows OmpF trimer cross-linked to one to three PL molecules. Similar lipid cross-linking profiles are observed for peak 1 of other OmpF^BPA^ mutants (figs. S10 and S11). Inset: a zoomed view of 21+ charge state. Observed masses are listed in table S4. Gel inset of the same sample stained with Coomassie and emerald green confirms the absence of LPS staining for OmpF V177^BPA^ peak 1. (**E**) Native-MS spectrum for OmpF V177^BPA^ from peak 2. Peak 2 corresponds to OmpF cross-linked to LPS with or without PLs and copurified FepA and FhuA (apo- and LPS-bound forms). Insets: a zoomed view of OmpF trimer 21+ charge state and SDS-PAGE gel of sample confirm the presence of LPS in the cross-linked sample.

Given the paucity of inter-OMP adducts, we surmised that cross-linking to lipid was the likely outcome. Discrete LPS binding sites have been identified crystallographically in several OMPs, including OmpF and TBDTs (*39*–*41*). We determined whether covalent BPA cross-links toward such LPS sites occurred in vivo using denaturing SDS-PAGE followed by staining with the LPS-specific fluorescent dye, Pro-Q emerald green. In agreement with previous structural studies, BtuB G455^BPA^ and V523^BPA^, FepA V484^BPA^ and OmpF V177^BPA^, V196^BPA^, G198^BPA^, and T238^BPA^ resulted in BPA-mediated cross-linking to LPS (Figs. 2 and 3 and tables S2 to S4). We also detected in vivo LPS cross-links to additional sites, including BtuB W164^BPA^ and G242^BPA^, FepA I255^BPA^ (Fig. 2), and OmpF L281^BPA^ (Fig. 3).

We exploited native-state mass spectrometry (native-MS) to establish lipid identities and stoichiometry for OMP^BPA^ cross-linked mutants (Fig. 3, D and E). Native-MS has previously identified a single LPS molecule bound to folded BtuB (*42*), which was replicated in the present work (fig. S7). A single LPS molecule was similarly identified bound noncovalently to wild-type FepA (fig. S7). In both cases, however, LPS-bound and unbound mass-to-charge ratio (*m/z*) peaks confounded attempts to define OMP^BPA^-lipid populations more precisely, other than occasional cross-links to PLs (e.g., BtuB G242^BPA^, BtuB G242^BPA^ G455^BPA^, and FepA V679^BPA^; figs. S8 and S9). By contrast, no lipids were bound to wild-type OmpF trimer in native-MS experiments (fig. S7). This observation, along with the greater yields of cross-linked OmpF^BPA^ mutants recovered from membranes, greatly simplified subsequent MS analysis (figs. S10 to S12). We found that UV-activated OmpF^BPA^ mutants eluted as two peaks from anion exchange chromatography, whereas wild-type OmpF eluted as a single peak with a shoulder (Fig. 3A). Anion exchange peaks for each UV-activated OmpF^BPA^ mutant were initially analyzed by SDS-PAGE and LPS staining, which indicated that peak 2 samples corresponded to LPS–cross-linked proteins (Fig. 3, B and C). Native-MS demonstrated that both peaks yielded distinct lipid signatures, exemplified by OmpF V177^BPA^ (Fig. 3, D and E). The OmpF V177^BPA^ spectrum from peak 1 showed binding of up to three PLs per trimer, whereas OmpF V177^BPA^ from peak 2 was cross-linked to LPS or combinations of LPS and PLs. Similar to wild-type OmpF, no lipids were detected bound to OmpF V177^BPA^ that had not been exposed to UV light (fig. S7), confirming that the PLs and LPS bound to the BPA mutants are covalently linked. The observed cross-linking of OmpF V177^BPA^ to either LPS or PLs is explained by its central location in the hydrophobic β barrel where the alkyl chains of both lipids meet. Other mid-barrel OmpF^BPA^ mutants (V196^BPA^, G198^BPA^, T238^BPA^, and L281^BPA^) yielded essentially identical results (fig. S10). In contrast to mid–β barrel mutants, OmpF sites located toward the periplasm (Y202^BPA^, Y242^BPA^, and Y285^BPA^), comprising the so-called “aromatic girdle,” resulted exclusively in PL cross-links (fig. S11). BPA incorporation sites toward the extracellular side of the OmpF β barrel (E234^BPA^, Q277^BPA^, and D312^BPA^) resulted in little or no detectable LPS (or PL) cross-linking (fig. S12). Molecular dynamics simulations suggested that the lack of reactivity is due to the low number of C─H bonds in the extracellular head group of LPS, in which BPA typically inserts (fig. S13A).

In summary, BPA-photoactivatable cross-linking into the OM of *E. coli* demonstrates that the predominant first-shell interactions of OMPs are to lipids, not to other OMPs. Our cross-linking approach is validated by the fact that the lipid asymmetries observed for OmpF reflect directly the asymmetric nature of the OM itself (fig. S13), first described by Kamio and Nikaido (*43*) in bulk modification experiments on *Salmonella*.

### Promiscuous OMP-OMP associations are mediated by interfacial lipids

A notable consequence of BPA-mediated OMP cross-linking to OM lipids was the copurification of other OMPs (Figs. 2, A and C, and 3, B and C), identified by LC-MS/MS of extracted bands from SDS–polyacrylamide gel electrophoresis (SDS-PAGE) gels and, in the case of OmpF and FepA, confirmed by native-MS experiments (Fig. 3, D and E, figs. S9 to S12, and tables S2 to S4). The migration positions of additional OMPs in SDS-PAGE, along with the absence of any peptides from the BPA-containing proteins themselves, indicated that they were not cross-linked species but had been retained during purification as a consequence of the target OMPs’ covalent attachment to OM lipids. This interpretation is given further weight by the observation that coeluting, detergent-solubilized OMPs are readily separated by ion exchange chromatography in the absence of BPA cross-linking (fig. S14). The same phenomenon was observed for all three target OMPs although the abundance, number, and identity of additional OMPs that copurified varied, sometimes even for different barrel positions within the same OMP. BtuB W164^BPA^, which engages in a direct protein-protein interaction with BamA (fig. S6), likely also associates with BamA via LPS (Fig. 2A), emphasizing the alternate ways by which promiscuous interactions are mediated. Several BtuB^BPA^ proteins copurified with small amounts of the peptidoglycan-binding protein OmpA, one of the most abundant proteins in the OM. The absence of OmpA from most of our experiments is likely due to its retention with the cell wall since lysozyme was not used in the OMP extraction protocol. FepA^BPA^ mutants resulted in the copurification of up to five additional OMPs. SDS-PAGE showed FepA^BPA^ mutants copurifying with the porins OmpF and OmpC, the siderophore transporters FhuE and FhuA, and the LPS insertase LptD (Fig. 2C). Fluorescence microscopy experiments showed that LtpD was clustered within OmpF-containing regions similar to the clustering observed for TBDTs (fig. S1F). Native-MS data further indicated that FepA coextracted porins are predominantly homo- and heterotrimers of OmpF and OmpC (fig. S9 and table S3) (*44*, *45*). Last, both SDS-PAGE and native-MS showed that most UV-activated OmpF^BPA^ mutants, particularly when cross-linked to LPS, resulted in copurification of FepA and FhuA (Fig. 3, figs. S10 to 12, and table S4).

**Fig. 4.**
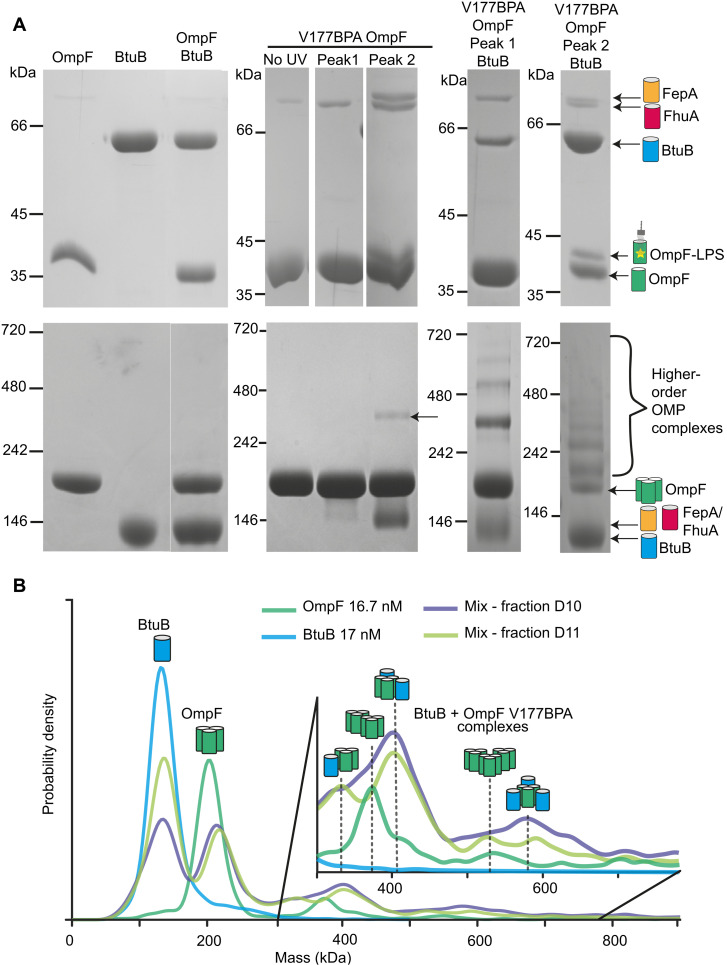
OM lipids mediate promiscuous higher-order complexes between OMPs in vitro. (**A**) Denaturing SDS-PAGE (top) and blue-native PAGE (bottom) of detergent-solubilized OmpF and BtuB confirm that no high–molecular weight complexes are detected in single protein controls and in a 1:1 mixture (20 μM protein concentration). OmpF V177^BPA^ without UV exposure resembles wild-type OmpF in both SDS-PAGE and native PAGE. Peak 1 and peak 2 of UV-exposed OmpF V177^BPA^ cross-linking to PL and LPS, respectively, copurify with monomeric OMPs FhuA and/or FepA. The abundance of FepA and FhuA in the peak 2 (LPS bound) sample is sufficient to detect a higher–molecular weight complex in blue-native PAGE demonstrating that cross-linked LPS promotes a promiscuous complex between trimeric OmpF and the copurified TBDTs (arrow). Addition of purified BtuB (15 μM, with LPS bound noncovalently; fig. S7) to OmpF V177^BPA^ peak 1 and peak 2 samples results in the appearance of higher-order complexes that are absent in the single protein controls. (**B**) Mass photometry data were collected following passage of OMPs through a size exclusion column (SEC). The average plot for the pooled elution peak fractions is shown for OmpF V177^BPA^ peak 1 (green, 17 nM) and BtuB (blue, 17 nM). Two discrete SEC fractions (D10 and D11) of the BtuB-OmpF V177^BPA^ mixture (pale green and purple) are also plotted. BtuB control data show that most of the species are monomeric with some dimer formation. Similarly, the OmpF V177^BPA^ peak 1 control data show some self-association of OmpF trimers. Comparison of these individual protein controls with data for BtuB and OmpF V177^BPA^ mixture reveals an increased abundance of high–molecular mass species relative to the control samples. The inset shows the assignment of OMP complexes within the sample to corresponding high mass peaks.

LPS-mediated, noncovalent complexes of UV-activated OmpF^BPA^ and FepA^BPA^ mutants with other OMPs do not survive gas phase energization in native-MS experiments. We therefore developed alternate approaches to investigate these higher-order assemblies in vitro, focusing on complexes of OmpF and the TBDTs BtuB and FepA. For these experiments, we used the mid-barrel mutant OmpF V177^BPA^, which cross-links to either PLs (peak 1) or LPS (peak 2). SDS-PAGE of these purified fractions showed a similar pattern to the previous experiments wherein cross-linking to LPS was more effective at extracting additional OMPs from the membrane (FepA/FhuA) than PL cross-links. Analyzing these fractions by blue native PAGE, an electrophoretic method for membrane protein complexes (*46*), revealed a heterologous complex of OmpF V177^BPA^-LPS bound to FepA or FhuA (Fig. 4A). We next ascertained whether OmpF V177^BPA^ cross-linked to PLs or LPS can form noncovalent complexes with exogenously supplied BtuB or FepA. The resulting high–molecular weight laddering in blue native PAGE—not observed in the no–cross-linking and single protein controls—was consistent with these OMPs forming noncovalent complexes with OmpF (Fig. 4A). Addition of purified LPS to samples amplified the laddering effect and demonstrated that all three OMPs weakly self-associate to form dimers or dimer of trimers for OmpF (fig. S15). We used mass photometry to estimate protein masses. Our analysis focused on OmpF V177^BPA^-PL (peak 1) due to monomeric barrel contamination in OmpF V177^BPA^-LPS (peak 2). Samples were mixed with BtuB (bound noncovalently to an LPS molecule), which revealed a range of complexes of varying mass. In control experiments, self-associated oligomers were observed, typically extending to a dimer for monomeric barrels or dimer of trimers for OmpF (Fig. 4B). However, in OmpF/BtuB mixtures, heterogeneous complexes predominated in mass photometry experiments, which showed OmpF associating with up to three BtuB molecules to form a ~600-kDa complex (Fig. 4B and fig. S16).

We conclude that promiscuous associations of OMPs in *E. coli* are mediated by the asymmetric lipids that surround them. The covalent attachment of first-shell lipids to OMPs, particularly LPS, significantly stabilized OMP-lipid-OMP complexes that otherwise dissociate during detergent solubilization of the OM. As a result, we uncovered a propensity for trimeric porins to associate with monomeric OMPs such as TBDTs.

### LPS promotes promiscuous associations between heterologous OMPs, suppressing mobility and mediating supramolecular lattices that maintain OM integrity

**Fig. 5.**
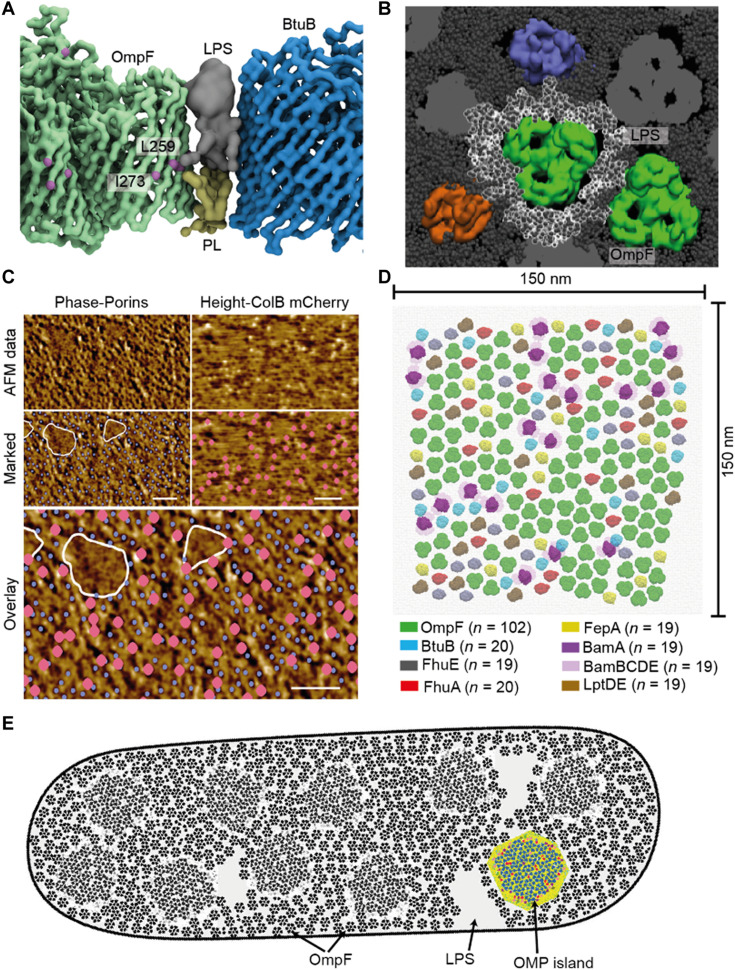
OMP-lipid-OMP complexes are the functional units of supramolecular OMP assemblies that stretch across the entire *E. coli* OM. (**A**) Snapshot of MD simulation for the OmpF-LPS/PL-BtuB complex showing the mutual sharing of asymmetric lipids. OmpF mid-barrel residues L259 and I273 (purple spheres) are highlighted for reference. (**B**) Snapshot of molecular dynamics (MD) simulation for a heterologous lipid-mediated complex formed between trimeric OmpF (green) and neighboring OMPs. LPS is shown in dark gray with molecules surrounding the central OmpF in white. (**C**) AFM imaging the OM of a live *E. coli* MG1655 cell labeled with FepA-binding ColB-mCherry (marked as pink balls). As in previous work (*30*), phase images provide the highest contrast for detecting the trimeric porin network (purple dots). The simultaneously recorded height image shows the positions of ColB-mCherry fluorescent labels as local height maxima. The overlay of FepA positions with the trimeric porins demonstrates that FepA is embedded within the porin network. In addition, there are regions where OMPs do not appear, previously identified as LPS-enriched domains (*30*). Scale bars, 50 nm. Color (phase/height) scales are 1.1°/2.5 nm and 1.1°, respectively. (**D**) Model of the simulated OMP island (SOI) in which OmpF (green) hosts heterologous OMPs within its hexagonal arrays. Monomeric β barrels and associated proteins incorporated into the island (detailed in key) were all identified in the present work. A total of 102 OmpF trimers are presented in the model, forming 18 hexagons. *n*, number of specific OMP in simulation. (**E**) Cartoon of an *E. coli* cell depicting the OMP-LPS-OMP network across the OM. The high relative abundance of OmpF results in the porin being spread over much of the cell surface. OMP islands (highlighted by colored OMP island) immersed within a black and white OMP background are embedded within the OmpF network.

To understand how heterologous OMP-lipid-OMP complexes lead to higher-order OMP assemblies, we developed a computational model of a supramolecular OMP island (Fig. 5), the structure and composition of which were based on cross-linking, native-MS, fluorescence microscopy, and AFM data. The model is founded on six principles/assumptions. First, most OMPs are surrounded by a shell of asymmetric lipids. Second, rather than residing within a sea of LPS, OMPs are often associated with other OMPs via interfacial lipids (Fig. 5A). Third, the network formed by the abundant porins OmpF/C dominates the OM landscape. Low-abundance OMPs such as TBDTs and LptD reside within these networks. Colocalization is supported by diffraction-limited epifluorescence data, where TBDTs and LptD reside in OmpF-rich regions (fig. S1), and by AFM data that identify individual FepA molecules within OmpF networks (Fig. 5C). Fourth, we suggest that “guest” OMPs such as TBDTs not only reside within porin-rich regions but also associate with these porins through shared annular lipids, likely exploiting the threefold symmetry of the porin (Figs. 4B and 5B). Fifth, previous in vitro and in vivo AFM data show that porins form imperfect hexagonal arrays interspersed with small triangular arrangements (fig. S17) (*30*, *47*–*50*). Our supramolecular model respects two aspects of these sixfold and threefold symmetries, the distance between OMP-OMP centroids (~80 to 90 Å) and the internal angles of the triangles (~57° to 63°). Sixth, OMP islands/clusters are likely to be diverse in terms of their composition, containing both monomeric and trimeric OMPs.

We incorporated seven different OMPs in a simulated OMP island (SOI); OmpF, BtuB, FhuE, FhuA, FepA, BamA (and lipoproteins, BamBCDE), and LptD (and lipoprotein LptE). OmpA was not included for the reasons discussed above. To make the SOI computationally tractable, its dimensions were set to 150 nm by 150 nm, which is similar to the dimensions reported for BamA clusters determined by three-dimensional structured illumination microscopy (*29*) but smaller than most estimates of OMP islands/clusters (*27*, *28*). The assumptions underpinning the SOI meant that it could accommodate 218 OMPs within its lattice structure. Of less importance was the absolute number for each OMP, with the exception of OmpF, the clustering of which is the basis for the lattice. Here, we applied the constraint that OmpF be present at 47% of the total, which is similar to whole-cell abundance estimates for OmpF/C from proteomic studies (*32*). The remainder was distributed equally among monomeric OMPs in the model. This resulted in abundance estimates for some OMPs (FepA and BtuB) that are consistent with estimates from photobleaching data (fig. S1), while others (e.g., BamA and LptD) are more speculative. The key point is that *E. coli* OMPs, regardless of β barrel size or relative abundance, are all accommodated within a common hexagonal array.

The OMPs of the SOI were incorporated into an asymmetric bilayer (*51*) made up of 8093 LPS and 25,099 PL molecules [90% 16:0 to 18:1 phosphoethanolamine (POPE), 5% 16:0 to 18:1 palmitoylphosphatidylglycerol (POPG), and 5% cardiolipin]. The form of LPS used in the computational model lacks O-antigen, commonly referred to as rough LPS. This form of LPS is the same as that found in the *E. coli* K-12 strains used in the present study (MG1655, BW25113, and BZB1107). LPS outnumbers OMPs by almost two orders of magnitude in the SOI. ~18 LPS molecules envelope an OmpF trimer, and ~13 LPS molecules envelope a TBDT β barrel. The total mass of the SOI is ~80 MDa, ~27% of which is accounted for by OMPs. To evaluate how good the computational model is of native OMP clustering in *E. coli*, we compared OMP near-neighbor contact distributions from the SOI with those from live-cell AFM data (*30*). The comparison demonstrates that the SOI is a reasonable representation of OMP clustering (fig. S17). Moreover, removal of intervening LPS (see below) generated near-neighbor contact distributions significantly smaller than those seen in vivo (fig. S17), which is consistent with these networks in *E. coli* being mediated by LPS. Further confirmation that the lattice arrangement of the SOI is a fair depiction of OMP clustering is the close apposition of separate BAM complexes, which is in agreement with recent inter-BAM^BPA^ cross-linking studies (*52*). Last, for very long (microsecond) simulations, we reduced the system yet further to a subsection of the SOI that contained 48 OMPs/4373 LPS/13,059 PLs.

The SOI was used to explore two aspects of this supramolecular assembly: its packing and internal mobility. Long simulations showed that interfacial PLs are mobile within the island. By contrast, OMPs and associated LPS molecules are largely immobile (movie S1). Both observations are consistent with experimental data even though the time domains for the latter are typically six orders of magnitude longer (*27*, *53*), emphasizing that even microsecond simulations capture the essence of differential mobility between the two lipid envelopes. The original OMP island hypothesis posited that direct OMP-OMP associations drive OMP clustering (*54*), yet in the present work, only 1 of 29 BPA mutants identified such a contact. We used the SOI to explore why these contacts are rare. Interfacial LPS and PLs were manually removed from the system, leaving “holes” in the OM. After 2 μs of simulation, some of the OMPs had moved to interact directly with each other, but holes between them remained, some the size of the antibiotic vancomycin (fig. S18). Thus, direct OMP-OMP packing is poor in the absence of LPS, potentially compromising the barrier function of the membrane.

Our cross-linking data suggest that although both lipids of the OM mediate promiscuous OMP associations, LPS is most effective (Fig. 4). We compared the outcomes of simulations for OMPs residing in either a symmetric PL/PL or asymmetric LPS/PL membrane to determine the basis for this difference. OMPs and PLs showed increased mobility in the symmetric membrane but, as in previous simulations, OMPs and LPS were static in the asymmetric membrane (fig. S19) (*55*). Consequently, the triangular and hexagonal lattice arrangements of OMPs were quickly lost in the symmetric PL bilayer (fig. S19). Closer examination of OMP-lipid lifetimes in the two simulations showed that LPS interacted with an OMP for 97.3% of the simulation, with essentially equal contributions from the three portions of the molecule (lipid tails, head group, and glycans). By contrast, PL interactions lasted for 5.6% of the simulation, the transient nature of the interaction due to their smaller size and reduced propensity for hydrophobic, polar, and electrostatic interactions. The SOI therefore explains why BPA-mediated cross-linking to LPS stabilizes interconnections between OMPs more effectively than PLs (Figs. 4 and 5 and fig. S19).

The dual hydrophobic/hydrophilic nature of LPS makes the OM impermeable to molecules of either polarity. The present work demonstrates that an additional advantage of LPS in the outer leaflet is its stabilization of OMP assemblies, which, by reducing lateral mobility, maintains the structural integrity of the OM.

## DISCUSSION

Less than 10% of the *E. coli* MG1655 OM is LPS only (*30*). The bulk of the OM instead comprises networks of OMPs, primarily porins (*30*). These regions are nevertheless heterogeneous, as shown by the presence of OMP islands within OmpF-rich regions (fig. S1). Together, a picture of the *E. coli* OM as a mosaic of heterogeneous OMP islands/clusters within an expanse of porins emerges (Fig. 5E). Why some OMPs (such as TBDTs) appear clustered on the cell surface, while OmpF is more widely distributed, remains an open question. The different patterns might reflect the same punctate secretion to the OM, for example, via a Sec-BAM supercomplex as has been proposed recently (*56*), but with distinct outcomes depending on levels of expression.

The basic organizational units of OMP networks are noncovalent OMP-LPS-OMP complexes. These units are the building blocks of larger assemblies in which low-abundance, monomeric OMPs are accommodated. Although the nature of the cross-linking experiments captures only direct OMP-LPS interactions, it is possible that more than one shell of LPS could contribute to the maintenance of OMP-LPS-OMP complexes. The resulting heterologous structures contain functionally diverse OMPs, including the biogenesis proteins LptD and BamA. Their presence ensures local deposition of LPS and OMPs, respectively, circumventing the problem of restricted diffusion (*54*). Collectively, OMP-LPS-OMP complexes likely constitute a formidable mode of cell envelope stabilization. For example, there are ~1400 OMP-LPS-OMP contacts in our 150 nm × 150 nm SOI model. Since the SOI constitutes ~0.06% OM surface area of a typical *E. coli* cell (~6 μm^2^), this equates to >10^6^ LPS-mediated cross-bridges interlinking the OMPs of the OM, which is in addition to the stabilization afforded by divalent cations that bridge the head groups of neighboring LPS molecules. We suggest that the OMP-LPS-OMP network explains many of the biophysical properties of the *E. coli* OM: the absence of long-range diffusion (*54*), its stiffness, load-bearing capacity (*57*, *58*), and its driving of the terminal stages of BamA-catalyzed OMP folding (*59*).

Supramolecular OMP organization sheds new light on several areas of OM biology: OM expansion: The impermeability of the OM has to be maintained during growth. Insulating every OMP from its neighbor by a layer of LPS overcomes this problem while still building a stabilizing lattice. OMP turnover: The LPS-mediated interaction network leads to the spatiotemporal behavior of OMPs, which, in turn, leads to their turnover by binary partitioning (*27*, *29*). Binary partitioning would be impossible in a symmetric PL/PL membrane because of protein mixing. Maintenance of OM asymmetry: If PLs inadvertently become localized to the outer leaflet, they are removed and shuttled back to the inner membrane by the maintenance of lipid asymmetry (Mla) system. MlaA is the starting point for retrograde transport through its selective extraction of PLs from the outer leaflet. MlaA binds OmpF and OmpC (*60*, *61*), but why this close association exists has been a mystery. The present work suggests that the rationale for this pairing may have its origins in protection of the LPS-mediated porin lattice, which, as a consequence, maintains lipid asymmetry. Colicin and bacteriophage intoxication: Colicins and bacteriophages typically associate with two components of the OM to activate import and infection, respectively, in *E. coli*. The long tail fibers of bacteriophage T4 bind LPS and OmpC (*62*), and colicin E9 binds OmpF and BtuB (*35*, *42*). In both cases, the two components are colocated by virtue of the lipid-mediated OMP network. Colocation has been observed directly in the OM translocon structure of colicin E9 wherein the β barrels of BtuB and OmpF abut each other, with unassigned density wedged between them likely to be intervening LPS (*67*). S-layer assembly: Many Gram-negative bacteria assemble a protein S-layer beyond the OM through noncovalent attachment to the oligosaccharides of smooth LPS. Recent structures of the *Caulobacter crescentus* S-layer protein RsaA bound to O-antigen reveal hexagonal arrays, but how these relate to the OM ~180 Å away on the cell surface is unclear (*63*, *64*). We speculate that the hexagonal arrangement of S-layer proteins may be mirroring sixfold symmetries intrinsic to the supramolecular organization of the OM itself.

## MATERIALS AND METHODS

### Colicin purification

Colicins were purified as described previously following expression in BL21 (DE3) cells New England Biolabs (NEB) and purified using nickel affinity chromatography and size exclusion chromatography (SEC) (*33*, *65*–*67*). In cases where conjugation of Alexa Fluor maleimide was required, disulfide bonds were reduced with dithiothreitol (DTT; 5 mM), following which the reducing agent was removed, by passage over a HiTrap high performance (HP) desalting column (5 ml, Cytiva). Alexa Fluor dye (10 mM in 100% dimethyl sulfoxide) was added to protein in threefold molar excess, and the sample was left to incubate in the dark at room temperature for 1 hour. The reaction was quenched with DTT (10 mM), and excess dye was removed by desalting. The desalted sample was applied to a Superdex 10/300 column equilibrated in 20 mM tris-HCl (pH 7.5) and 150 mM NaCl, and protein-containing fractions were pooled. Final protein concentration and labeling efficiency was determined by UV absorbance using sequence-based extinction coefficient and dye absorbance coefficients provided by Thermo Fisher Scientific.

### Microscopy

A day before microscopy, *E. coli* MG1655, a K-12 strain with a truncated form of LPS, was grown in 10 ml of LB and then transferred to M9 glucose [M9 minimal media, 2 mM MgSO_4_, 0.1 mM CaCl_2_, 0.4% (w/v) glucose, and 0.05% casamino acids] overnight. On the morning of microscopy, a 500-μl aliquot of overnight culture was pelleted and resuspended in 4 ml of fresh M9 glucose. *E. coli* MG1655 was grown to an OD_600_ (optical density at 600 nm) of 0.4 to 0.5. OD_600_ 0.6 culture (500 μl) was pelleted and resuspended in 200 μl of fresh M9 glucose supplemented with 200 nM ColN^1-185^mCherry and 200 nM ColE9^AF488^ (for BtuB) or 200 nM ColB-GFP (for FepA). Labeling was conducted for 10 min, at room temperature, on a rotary shaker. Labeled cells were pelleted and resuspended in 1 ml of 4% formaldehyde at 4°C for 30 min [diluent: phosphate-buffered saline (PBS)]. After fixing, three wash steps were conducted in PBS. Aliquots (4 μl) of cells were loaded onto 1% PBS-agarose pads for imaging. Imaging was conducted using an Oxford Nanoimager S with a 100× 1.49 numerical aperture (NA) oil immersion objective. ColN^1-185^mCherry-labeled OmpF was visualized with a 561-nm laser line at approximately 20 mW. ColE9AF488-labeled BtuB and ColB-GFP–labeled FepA were visualized with a 473-nm laser line at approximately 20 mW. Imaging was conducted at an exposure time of 100 ms.

### OmpF and LptD colabeling

A day before microscopy, *E. coli* BW25113, a K12 strain with a truncated form of LPS, was grown in 10 ml of LB and then transferred to M9 glucose [M9 minimal media, 2 mM MgSO_4_, 0.1 mM CaCl_2_, 0.4% (w/v) glucose, and 0.05% casamino acids] overnight. The following morning, a 500-μl aliquot was pelleted and resuspended in 4 ml of fresh M9 glucose. *E. coli* BW25113 was grown to an OD_600_ of 0.4 to 0.5. Cells were fixed by exposure to 4% formaldehyde at 4°C for 30 min. Fixed cells were pelleted and resuspended in 4 ml of PBS supplemented with 0.3% Triton X-100 for 20 min, at which point 500 μl of culture (OD_600_ of 0.6) was pelleted and resuspended in 100 μl of PBS supplemented with 0.01% Triton X-100, 200 nM Colicin N^1-185^mCherry, and AF488 Fab LptD antibody (0.038 mg/ml; 2B11) (*68*). Labeling was carried out for 1 hour, at room temperature on a rotary shaker followed by three wash steps in PBS + 0.01% Triton X-100. Aliquots (4 μl) of cells were loaded onto PBS, 1% agarose pads for imaging. Imaging was conducted as described above. A calibration bead slide was imaged for channel alignment.

#### Maxima per cell analysis

The long axis, short axis, and intensity of each cell were normalized, to ensure that intensity-independent fluorescence distribution was directly comparable between cells. Each cell image was converted to 8-bit grayscale, and maxima were identified with the “find maxima” ImageJ tool set to a prevalence of 20. Maxima found outside the bounds of cells were eliminated.

#### Intensity based protein counting

Cells were prepared as described above. For each sample, 10 fields of view of 1000 frames were collected at an exposure time of 50 ms. Bleaching was conducted with a 20-mW 473-nm laser. The intensity of single ColE9^AF488^ and ColB-GFP proteins was determined by measuring the intensity of blinking molecules toward the end of the 1000 frame acquisitions. The average fluorescence intensity of the off and on state of blinking molecules was determined. The following equation was used to estimate the number of proteins per island

$$\text{Proteins per island}=\frac{I_{i}-B_{i}}{F_{i}}$$

where *I_i_* is the unbleached protein island intensity, *B_i_* is the background (off state) intensity of a single blinking fluorophore, and *F_i_* is the intensity of a single blinking fluorophore.

### BPA incorporation and extraction of OM fractions

Plasmids encoding *ompF*, *btuB*, and *fepA* were modified by site-directed mutagenesis to incorporate TAG amber stop codon at selected sites. Following transformation of each mutated plasmid along with the pVol plasmid into chemically competent cell lines BZB1107 (Δ*ompF*, Δ*ompC*, and Δ*lamB*) for OmpF, RK5016 (*metE70 argH btuB recA*) for BtuB, and BW25113 (Δ*fepA*) for FepA. All cell lines used for protein expression have truncated LPS molecules; BZB1107 is a B strain with an insertion sequence (IS) element in the *waaT* gene (*69*), while RK5016 and BW25113 K-12 strains have an IS5 insertion in the *wbbL* gene (*70*–*72*). Cells were grown in LB (pH 6.4), containing 0.5 mM BPA (dissolved in 0.8 M NaOH and pH maintained by the addition of 0.8 M HCl) and appropriate antibiotics, until an OD_600_ of 0.5 to 0.7 was attained. Protein expression was induced through addition of 0.15% arabinose, and OmpF-producing cells were maintained at 37°C for 2 hours or maintained at 18°C overnight for BtuB- and FepA-producing cells. Cells were harvested by centrifugation (4200 rpm, 20°C, 12 min) and resuspended in 2 ml of lysis buffer [10 mM tris-HCl (pH 8.0), 0.25% lithium 3,5-diiodosalicylic acid, and 1 mM phenylmethylsulfonyl fluoride per liter of cells]. Cells (5 to 8 ml) were added to a 10 cm by 10 cm petri dish on an ice pack in a CL-1000 Ultraviolet Crosslinker and exposed to UV (365 nm) for 90 min, with mixing at 45 min. OM fractions containing OmpF, BtuB (*42*), or FepA (*66*) were extracted following lysis by sonication (no lysozyme was added; thus, PG-interacting proteins remained insoluble) and through a series of centrifugation steps, with solubilization in 2% β-OG (*42*, *67*).

### Purification of OmpF and FepA

OM fraction containing OMP of interest was syringe-filtered (0.45 μm) and applied to new Q Sepharose resin [~20 ml = 1 column volume (CV)] equilibrated in 10 mM tris-HCl (pH 8.0), 5 mM EDTA, and 1% β-OG. After two CV washes, protein was eluted using a linear salt gradient from 0 to 500 mM LiCl across 3.6 or 4.5 CV, for OmpF and FepA, respectively. Fractions containing the protein of interest, as determined by SDS-PAGE, were pooled and concentrated to <2 ml before loading on a Superdex 200 16/60 column equilibrated in 10 mM tris-HCl (pH 8.0), 5 mM EDTA, and 1% β-OG. Buffer-exchanged protein was syringe-filtered (0.45 μm) and applied to a MonoQ 4.6/100 PE column equilibrated in SEC buffer. Following a four CV wash step, the protein of interest was eluted using a linear salt gradient from 0 to 400 mM LiCl over 14 or 30 CV for OmpF and FepA, respectively. SDS-PAGE analysis was used to confirm the identity of OMPs within each elution peak. Individual peaks were pooled separately, spin-concentrated (50,000 molecular weight cutoff (MWCO), vivaspin), and desalted using a HiTrap 5-ml desalting column. Protein concentration was estimated from absorbance at 280 nm assuming a sequence-based extinction coefficient of 54,210 M^−1^ cm^−1^ for OmpF, 12,083 M^−1^ cm^−1^ for BPA, and 156,315 M^−1^ cm^−1^ for FepA.

### Purification of BtuB

Solubilized OM fraction was syringe-filtered (0.45 μm) and loaded onto a new diethylaminoethanol Sepharose resin packed in an XK 16/20 column (~20 ml CV), equilibrated with 90% low-salt buffer [50 mM tris-HCl (pH 7.5), 5 mM EDTA, and 0.54% β-OG] and 10% high-salt buffer [50 mM tris-HCl (pH 7.5), 5 mM EDTA, 0.54% β-OG, and 1 M LiCl]. The column was washed with 10% high-salt buffer until a stable baseline was obtained (~2 CV), and further contaminants were removed by a 10 to 50% high-salt buffer linear gradient over 3.5 CV. BtuB was eluted in 1-ml fractions (96-well plate) after stepping to 100% high-salt buffer. The sample was left overnight at 4°C, during which BtuB precipitated. BtuB was pelleted by centrifugation (12,000*g*, 4°C, 15 min) and resuspended in SEC buffer containing 10 mM tris-HCl (pH 8.0), 5 mM EDTA, and 1% β-OG. Protein was loaded onto a Superdex 200 16/60 column equilibrated in SEC buffer, and fractions containing BtuB, as confirmed by SDS-PAGE, were pooled. A final ion exchange step (MonoQ 4.6/100 PE column), followed by desalting, was applied where required, using the protocol described above for OmpF. Protein concentration was estimated from absorbance at 280 nm assuming a sequence-based extinction coefficient of 150,010 M^−1^ cm^−1^ for BtuB and 12,083 M^−1^ cm^−1^ for BPA.

### Colicin-based plate-killing assay

OM insertion of OMPs was validated through colicin-based killing assays. Cells (strains as described above for protein expression) were transformed with plasmid encoding the protein of interest and pEVOL-pBpF (Addgene plasmid #31190) (for BPA mutants only), and 5 ml of cultures grown to an OD_600_ of 0.6, at which protein expression was induced with 0.15% arabinose. Cells were subsequently maintained at 37°C for 2 hours before pelleting through centrifugation (4200*g*, 20°C, 5 min). Pelleted cells were resuspended in 0.2 ml of LB, mixed with 0.7% molten LB/agar (50°C), and poured on 1% LB/agar plates. All LB agar contained 0.5 mM BPA (dissolved in 0.8 M NaOH and pH maintained by the addition of 0.8 M HCl) and appropriate antibiotics. Colicin (2 μl) was spotted on LB agar plates and left to incubate overnight at 37°C. Colicin-based killing was indicated by areas of clearance in the bacterial lawn.

### Liquid growth colony-forming unit determination

Cells from overnight LB culture (5 ml) were used to inoculate 50 ml of LB supplemented with kanamycin (50 μg/ml), as required, and grown at 37°C until an OD_600_ of 0.5 to 0.7 was reached. Cells (25 ml) were transferred into a 10-cm petri dish and placed on an ice block in CL-1000 Ultraviolet Crosslinker. Cells were exposed to UV (365 nm) for a total of 150 min, removing a sample (1 ml) at 30-min intervals. A dilution series from 10^4^ to 10^11^ was made up in LB for each sample and 5 to 10 μl plated on 1% LB agar plates supplemented with kanamycin (50 μg/ml) as appropriate. Plates were left to grow overnight at 37°C. Colonies were counted and log colony-forming units (CFUs) were calculated for each time point. Final logCFUs values are the average of three biological replicates.

### SDS-PAGE and pro-Q emerald LPS staining

Proteins were diluted 1:4 with 4× SDS-loading dye containing 200 mM tris-HCl (pH 6.8), 8% (w/v) SDS, 0.4% (w/v) bromophenol blue, 40% (v/v) glycerol, and 400 mM β-mercaptoethanol. Samples were denatured by boiling at 98°C for 2 min and loaded (8 μl) into a 10% (w/v) bis-acrylamide gel. Gels were run in buffer containing 10% SDS, 250 mM tris-HCl, and 1.9 M glycine, at constant amplitude (30 mA) until dye front reached bottom of gel (~35 min). For SDS-PAGE, gels were stained with 10% (v/v) acetic acid, 50% (v/v) ethanol, and 0.2% (w/v) Coomassie blue R-250 for 30 min, and de-stained with 10% (v/v) acetic acid and 10% (v/v) ethanol. For LPS staining, gels were rinsed with water before fixing with 100 ml of 50% (v/v) methanol and 5% (v/v) acetic acid solution and mixed with gentle agitation (50 rpm orbital shaker) for 45 min. Fixation step was repeated with fresh solution. Following fixation, gels were washed with 100 ml of 3% (v/v) acetic acid and gentle agitation for 20 min; this was repeated twice before oxidizing LPS with 25 ml of periodic acid (Thermo Fisher Scientific, P30636) and incubating for 30 min with mixing. Gels were washed 3× with 3% acetic acid as described above. Pro-Q Emerald 300 stain solution was prepared fresh with 500 μl of stock (Thermo Fisher Scientific, P30635) diluted in 25 ml of staining buffer (Thermo Fisher Scientific, P30636). Gels were stained in the dark with gentle agitation (50 rpm) for 120 min. Wash step with 3% acetic acid was repeated twice as described above, before imaging fluorescence with GBOXCHEMI-XRQ gel box equipped with GeneSys software. After imaging for LPS fluorescence, gels were stained and destained with conventional SDS-PAGE reagents.

### Blue-native PAGE

Proteins were prepared (9 μl) at the required concentration (10 to 20 μM) in 10 mM tris-HCl (pH 8.0), 5 mM EDTA, and 1% β-OG buffer, to which 0.45 μl of 5% G250 (Thermo Fisher Scientific, BN2004) and 3 μl of 4× NativePAGE sample loading buffer (Thermo Fisher Scientific BN2003) were added. Running buffer (1×) was made from 20× stock of NativePAGE running buffer (Thermo Fisher Scientific, BN2001), and 1× light blue cathode buffer was made from 20× stocks of NativePAGE running buffer and Native PAGE cathode additive (Thermo Fisher Scientific, BN2002). NativePAGE Novex 4 to 16% bis-tris protein gel (Thermo Fisher Scientific, BN1002BOX) and buffer solutions were cooled to 4°C before loading samples (8 μl) and native mark unstained protein ladder (Thermo Fisher Scientific, LC0725). Gels were run at 4°C at 150 V for 1 hour, followed by 1 hour at 250 V. Gels were stained as described above for SDS-PAGE gels, with staining duration increased to 1 hour.

### LC-MS/MS cross-linking analysis

Purified OMPs were separated on 10% (acrylamide) SDS-PAGE gel and stained with Coomassie. Following destaining, gel bands were extracted individually, treated with 10 mM tris(2-carboxyethyl) phosphine, followed by 50 mM 2-chloroacetamide, and dried with 100% acetonitrile. Peptides were generated after digestion with 5 μg of sequencing-grade trypsin (Promega) at 37°C overnight. Peptides were analyzed on a Q Exactive mass spectrometer, as described previously (*66*, *73*). For protein ID experiments, data were searched against an *E. coli* database in MaxQuant (*74*). After confirming likely proteins within each gel band, the MS data were searched using the pLink software (*75*), with two missed cleavages, carbamidomethyl-Cys as fixed modification and Glu to pyro-Glu as variable modification. In this instance, the database searched contained the identified and target proteins and common contaminants. Data were initially filtered to a false discovery rate of 1% after which fragmentation patterns were used to isolate cross-links.

### Native mass spectrometry

Proteins (10 to 100 μM) in 10 mM tris-HCl, 5 mM EDTA, and 1% β-OG buffer were exchanged into an “MS buffer” containing 1% β-OG and 0.2 M ammonium acetate (pH 6.9) using a micro-biospin 6 column (Bio-Rad) according to the manufacturer’s manual. Each sample was diluted with the MS buffer to a final protein concentration of 5 μM before measurement. Mass spectra were acquired on a Q-Exactive hybrid quadrupole-Orbitrap mass spectrometer (Thermo Fisher Scientific, Bremen, Germany) optimized for transmission and detection of high–molecular weight protein complexes (*76*). An approximately 3-μl aliquot of the sample was transferred into gold-coated borosilicate capillary (Harvard Apparatus) and mounted on the nano electrospray ionization source. The instrument settings were 1.2-kV capillary voltage, S-lens RF 200%, argon UHV pressure 3.1 × 10^−10^ mbar, and a capillary temperature of 200°C. Voltages of the ion transfer optics—injection flatapole, inter-flatapole lens, bent flatapole, and transfer multipole—were set to 5, 3, 2, and 30 V, respectively. The noise level was set at 3. Protein ions were activated with a collisional activation voltage of 150 to 250 V in the HCD cells, without in-source trapping. Data were visualized and exported for processing using the Qual browser of Xcalibur 4.2 (Thermo Fisher Scientific).

### Mass photometry

Where applicable, OmpF V177^BPA^ was mixed with BtuB in a buffer containing 10 mM tris-HCl (pH 8.0), 5 mM EDTA, and 1% β-OG, in a 1:2 molar ratio and left at 4°C overnight to allow for complex formation. Individual proteins or protein mixtures (0.2 to 0.4 mg) were then transferred into amphipol for analysis using a protocol described previously (*67*). Samples were loaded onto SEC columns (Superose 6 increase 10/300), and individual fractions were collected (0.25 ml). Protein concentration in fractions was calculated from 280-nm absorbance using sequence-based extinction coefficients. Coverslips (no. 1.5, 24 × 50 and 24 × 24 mm^2^; VMR) were cleaned by sonication in 50% isopropanol (high-performance liquid chromatography grade) and Milli-Q water and oven-dried at 110°C for 1 hour. Measuring chambers were assembled by placing 2 × 2 microwell gaskets onto the cleaned coverslips. Measurements were taken using either a commercial mass photometer (One^MP^, Refeyn Ltd., Oxford, UK) or a similar, home-built mass photometer, as described previously (*77*). For each measurement, 3 μl of buffer solution was added to the gasket, and the focus position was identified and secured for the entire measurement. Sample (27 μl) freshly diluted to 30 to 60 nM was then added, and particle landing was recorded for 60 s. Fivefold frame binning and 4 × 4 pixel binning was applied during recording, resulting in a final frame rate of 200 Hz and a pixel size of 84.4 nm/pixel for the home-built and 70.2 nm/pixel for the commercial mass photometer. Each measurement was repeated at least three times, in separate gaskets.

The videos of landing assays were analyzed using DiscoverMP (version 2.3, Refeyn Ltd), which detects particle-binding events and calculates the interferometric scattering contrasts, with two user-specified image thresholds (T1 and T2) and number of frames averaged for continuous background removal, *n*_avg_, applied. For T1, *T* test of pixel density fluctuation due to sharp change of pixel intensity caused by particle binding to the glass surface is calculated, and the T1 value specifies the smallest amplitude change exceeding random noise associated with landing events. T2 measures the radial symmetry of the pixel neighborhood and specifies the lowest amount of radial symmetry expected at the center of a landing event, thanks to the radial symmetry signatures of landing events in interferometric images (*78*). Peak fitting is performed on groups of pixels that surpass both thresholds T1 and T2. The interferometric peak contrast was estimated using the amplitude of the peak fit. Analyzed lists of particles were exported, and data are analyzed and fitted using the MATLAB (2020b) curve-fitting toolbox.

To convert peak contrast to molecular weight values, we performed calibration measurements with proteins of known molecular weight. Internal standard with known oligomeric distribution was measured before the start and after the end of each set of measurements and analyzed using DiscoverMP as above, with peak centers fitted by DiscoverMP Gaussian fittings. The peak center values were then plotted against the known molecular masses of the protein standard and fitted to a curve *y = bx*, with *y* as contrast, *x* as mass, and *b* as calibration factor.

### Atomic force microscopy

BW25113 *E. coli* were grown in LB overnight, then diluted 100× to fresh LB, and grown for a further 2.5 hours. Exponentially growing cells were washed three times by spinning for 1 min at 7000*g* and resuspending in MM (1× M9 salts, 2 mM MgSO_4_, 0.1 mM CaCl_2_, and 0.4% glucose) at an OD_600_ of 0.5. Bacteria (500 μl) were then spun, resuspended in MM with 0.2 mM ColB-mCherry, incubated at room temperature on a rotary shaker for 10 min, and then washed with MM three times by spinning and resuspending. Last, cells were resuspended in 100 μl of 20 mM Hepes, applied to a Vectabond-coated coverslip for 5 min, and washed three times with 1 ml of MM. Vectabond-coated coverslips were prepared by thoroughly cleaning and then coating 13-mm glass coverslips (VWR) by sonicating in 1% SDS solution in a bath sonicator at 37 kHz and 100% power for 10 min, rinsing in milliQ water, and then ethanol and nitrogen drying; they were then plasma-cleaned in air at 70% power for 2 min. This cleaning process was then repeated once more. Clean coverslips were then coated by soaking in a 50:1 solution of acetone:vectabond (Vector Laboratories) for 5 min, and they were then rinsed in milliQ water, dried with nitrogen, and glued to clean glass slides (Reprorubber thin pour, Flexbar) (*79*).

Dynamic (AC) mode AFM was performed with a FastScanD cantilever on a Nanowizard III AFM with UltraSpeed head (Bruker AXS) with an Andor Zyla 5.5 USB3 fluorescence camera on an OlympusIX 73 inverted optical microscope. Dead cells were stained with SYTOX green nucleic acid stain (Thermo Fisher Scientific) and not used for imaging. The cantilever was driven at a frequency between 90 and 140 kHz, with a setpoint of 5 to 15 nm (corresponding to 50 to 70% of the free amplitude). Images were acquired at 500 nm and 512-pixel square, recorded at 2- to 8-Hz line frequency. Height and phase images were first processed by converting jpk files to text in gwyddion, a bandpass filter (1 to 50 px) was then applied in ImageJ (*80*) to remove the curvature of the cell, and a 1-pixel Gaussian filter was applied to remove high-frequency noise. Images were further processed using a custom macro that applied a bandpass filter (1 to 20 px) to remove general surface roughness and then the Find Maxima function was applied to find pores or labels from the phase and height channels, respectively, based on analysis code previously used and published elsewhere (*30*). Because of differences in contrast, pore finding was not always accurate; thus, missed or incorrect locations were corrected by visual inspection of the data and pore localizations. Patches were identified by hand. Color scales were set in gwyddion (*81*).

### Molecular dynamics simulations

The structures of the OMPs simulated were obtained from the RCSB protein databank: OmpF [Protein Data Bank (PDB): 3POX] (*82*), FhuA (PDB: 1QFG) (*41*), BtuB (PDB: 3M8D) (*83*), FepA (PDB: 1FEP) (*84*), FhuE (PDB: 6E4V) (*85*), LptDE (PDB: 4RHB), and BamABCDE (PDB: 5AYW) (*86*). To build the coarse-grained systems of the subsection of SOI embedded in a membrane, the Martini Maker tool (*87*) from the CHARMM-GUI server (*88*, *89*) was used, whereas the whole SOI system (150 × 150 nm) was built with the insane tool (*90*). The Martini force fields have previously been shown to reproduce experimental LPS, OMP, and PL behaviors and are the industry standard coarse-grained models for bacterial membrane simulations (*26*, *36*, *91*). Three systems (OmpF + three OMPs, OMP island, and smaller OMP island) were embedded in an OM model composed of a 100% rough LPS (Lacking O-antigen) outer leaflet and an inner leaflet containing 90% 16:0 to 18:1 POPE, 5% 16:0 to 18:1 POPG, and 5% cardiolipin. Explicit water (standard Martini) and counterion (0.2 M Na^+^ Cl^−^) particles were added to all simulations. Ca^2+^ ions were used to neutralize LPS negative charges. Molecular dynamics simulations were performed on the OmpF + three OMPs and on the smaller OMP island systems using the GROMACS simulation suite (version 2020.4) (*92*) along with the Martini 2.2 force field (*93*). The velocity rescale thermostat (*94*) with a coupling constant of *t* = 1 was applied to maintain the temperature constant at 313 K. The Parrinello-Rahman barostat (*95*) with a time constant of 12 ps was used for the pressure coupling. In the lipid removal equilibration simulations, lipids were removed in small batches, and then the system was equilibrated in steps of 20 ns initially, and 100 ns after the removal process was finished, in a total of 500 ns of equilibration. Overall, the simulation protocol followed the details provided in a previous report (*40*), with the total time for simulations being 2000 ns. Residence time interactions were calculated using the PyLipID software (*96*), using a dual cutoff of 0.475 and 0.8 nm.

## References

[1] C. J. Murray, K. S. Ikuta, F. Sharara, L. Swetschinski, G. Robles Aguilar, A. Gray, C. Han, C. Bisignano, P. Rao, E. Wool, S. C. Johnson, A. J. Browne, M. G. Chipeta, F. Fell, S. Hackett, G. Haines-Woodhouse, B. H. Kashef Hamadani, E. A. P. Kumaran, B. McManigal, R. Agarwal, S. Akech, S. Albertson, J. Amuasi, J. Andrews, A. Aravkin, E. Ashley, F. Bailey, S. Baker, B. Basnyat, A. Bekker, R. Bender, A. Bethou, J. Bielicki, S. Boonkasidecha, J. Bukosia, C. Carvalheiro, C. Castañeda-Orjuela, V. Chansamouth, S. Chaurasia, S. Chiurchiù, F. Chowdhury, A. J. Cook, B. Cooper, T. R. Cressey, E. Criollo-Mora, M. Cunningham, S. Darboe, N. P. J. Day, M. de Luca, K. Dokova, A. Dramowski, S. J. Dunachie, T. Eckmanns, D. Eibach, A. Emami, N. Feasey, N. Fisher-Pearson, K. Forrest, D. Garrett, P. Gastmeier, A. Z. Giref, R. C. Greer, V. Gupta, S. Haller, A. Haselbeck, S. I. Hay, M. Holm, S. Hopkins, K. C. Iregbu, J. Jacobs, D. Jarovsky, F. Javanmardi, M. Khorana, N. Kissoon, E. Kobeissi, T. Kostyanev, F. Krapp, R. Krumkamp, A. Kumar, H. H. Kyu, C. Lim, D. Limmathurotsakul, M. J. Loftus, M. Lunn, J. Ma, N. Mturi, T. Munera-Huertas, P. Musicha, M. M. Mussi-Pinhata, T. Nakamura, R. Nanavati, S. Nangia, P. Newton, C. Ngoun, A. Novotney, D. Nwakanma, C. W. Obiero, A. Olivas-Martinez, P. Olliaro, E. Ooko, E. Ortiz-Brizuela, A. Y. Peleg, C. Perrone, N. Plakkal, A. Ponce-de-Leon, M. Raad, T. Ramdin, A. Riddell, T. Roberts, J. V. Robotham, A. Roca, K. E. Rudd, N. Russell, J. Schnall, J. A. G. Scott, M. Shivamallappa, J. Sifuentes-Osornio, N. Steenkeste, A. J. Stewardson, T. Stoeva, N. Tasak, A. Thaiprakong, G. Thwaites, C. Turner, P. Turner, H. R. van Doorn, S. Velaphi, A. Vongpradith, H. Vu, T. Walsh, S. Waner, T. Wangrangsimakul, T. Wozniak, P. Zheng, B. Sartorius, A. D. Lopez, A. Stergachis, C. Moore, C. Dolecek, M. Naghavi, Global burden of bacterial antimicrobial resistance in 2019: A systematic analysis. Lancet 399, 629–655 (2022). 35065702 10.1016/S0140-6736(21)02724-0 PMC8841637

[2] ECDC/EMEA Joint Working Group, *The Bacterial Challenge: Time to React: A Call to Narrow the Gap Between Multidrug-Resistant Bacteria in the EU and the Development of New Antibacterial Agents* (EUR-OP, 2009).

[3] H. Nikaido, Molecular basis of bacterial outer membrane permeability revisited. Microbiol. Mol. Biol. Rev. 67, 593–656 (2003). 14665678 10.1128/MMBR.67.4.593-656.2003 PMC309051

[4] A. H. Delcour, Outer membrane permeability and antibiotic resistance. Biochim. Biophys. Acta 1794, 808–816 (2009). 19100346 10.1016/j.bbapap.2008.11.005 PMC2696358

[5] W. C. Wimley, The versatile beta-barrel membrane protein. Curr. Opin. Struct. Biol. 13, 404–411 (2003). 12948769 10.1016/s0959-440x(03)00099-x

[6] N. Noinaj, M. Guillier, T. J. Barnard, S. K. Buchanan, TonB-dependent transporters: Regulation, structure, and function. Annu. Rev. Microbiol. 64, 43–60 (2010). 20420522 10.1146/annurev.micro.112408.134247 PMC3108441

[7] F. Samsudin, M. L. Ortiz-Suarez, T. J. Piggot, P. J. Bond, S. Khalid, OmpA: A flexible clamp for bacterial cell wall attachment. Structure 24, 2227–2235 (2016). 27866852 10.1016/j.str.2016.10.009

[8] L. Vandeputte-Rutten, R. A. Kramer, J. Kroon, N. Dekker, M. R. Egmond, P. Gros, Crystal structure of the outer membrane protease OmpT from *Escherichia coli* suggests a novel catalytic site. EMBO J. 20, 5033–5039 (2001). 11566868 10.1093/emboj/20.18.5033 PMC125623

[9] V. Koronakis, A. Sharff, E. Koronakis, B. Luisi, C. Hughes, Crystal structure of the bacterial membrane protein TolC central to multidrug efflux and protein export. Nature 405, 914–919 (2000). 10879525 10.1038/35016007

[10] T. R. D. Costa, C. Felisberto-Rodrigues, A. Meir, M. S. Prevost, A. Redzej, M. Trokter, G. Waksman, Secretion systems in Gram-negative bacteria: Structural and mechanistic insights. Nat. Rev. Microbiol. 13, 343–359 (2015). 25978706 10.1038/nrmicro3456

[11] A. Du Toit, The T3SS translocon in biofilm formation. Nat. Rev. Microbiol. 13, 2–3 (2015). 10.1038/nrmicro3403 25417658

[12] T. Wu, J. Malinverni, N. Ruiz, S. Kim, T. J. Silhavy, D. Kahne, Identification of a multicomponent complex required for outer membrane biogenesis in *Escherichia coli*. Cell 121, 235–245 (2005). 15851030 10.1016/j.cell.2005.02.015

[13] N. Noinaj, A. J. Kuszak, J. C. Gumbart, P. Lukacik, H. Chang, N. C. Easley, T. Lithgow, S. K. Buchanan, Structural insight into the biogenesis of β-barrel membrane proteins. Nature 501, 385–390 (2013). 23995689 10.1038/nature12521 PMC3779476

[14] R. Voulhoux, M. P. Bos, J. Geurtsen, M. Mols, J. Tommassen, Role of a highly conserved bacterial protein in outer membrane protein assembly. Science 299, 262–265 (2003). 12522254 10.1126/science.1078973

[15] T. Wu, A. C. McCandlish, L. S. Gronenberg, S. S. Chng, T. J. Silhavy, D. Kahne, Identification of a protein complex that assembles lipopolysaccharide in the outer membrane of *Escherichia coli*. Proc. Natl. Acad. Sci. U.S.A 103, 11754–11759 (2006). 16861298 10.1073/pnas.0604744103 PMC1544242

[16] S. Qiao, Q. Luo, Y. Zhao, X. C. Zhang, Y. Huang, Structural basis for lipopolysaccharide insertion in the bacterial outer membrane. Nature 511, 108–111 (2014). 24990751 10.1038/nature13484

[17] S. Okuda, D. J. Sherman, T. J. Silhavy, N. Ruiz, D. Kahne, Lipopolysaccharide transport and assembly at the outer membrane: The PEZ model. Nat. Rev. Microbiol. 14, 337–345 (2016). 27026255 10.1038/nrmicro.2016.25 PMC4937791

[18] H. Nikaido, M. Vaara, Molecular basis of bacterial outer membrane permeability. Microbiol. Rev. 49, 1–32 (1985). 2580220 10.1128/mr.49.1.1-32.1985 PMC373015

[19] M. Vaara, Agents that increase the permeability of the outer membrane. Microbiol. Rev. 56, 395–411 (1992). 1406489 10.1128/mr.56.3.395-411.1992 PMC372877

[20] J. Kubiak, J. Brewer, S. Hansen, L. A. Bagatolli, Lipid lateral organization on giant unilamellar vesicles containing lipopolysaccharides. Biophys. J. 100, 978–986 (2011). 21320442 10.1016/j.bpj.2011.01.012 PMC3037713

[21] L. A. Clifton, M. W. A. Skoda, A. P. le Brun, F. Ciesielski, I. Kuzmenko, S. A. Holt, J. H. Lakey, Effect of divalent cation removal on the structure of Gram-negative bacterial outer membrane models. Langmuir 31, 404–412 (2015). 25489959 10.1021/la504407v PMC4295546

[22] L. A. Clifton, S. A. Holt, A. V. Hughes, E. L. Daulton, W. Arunmanee, F. Heinrich, S. Khalid, D. Jefferies, T. R. Charlton, J. R. P. Webster, C. J. Kinane, J. H. Lakey, An accurate in vitro model of the *E. coli* envelope. Angew. Chem. Int. Ed. Engl. 54, 11952–11955 (2015). 26331292 10.1002/anie.201504287 PMC4600229

[23] S. Lee, H. Bayley, Reconstruction of the Gram-negative bacterial outer-membrane bilayer. Small 18, 2200007 (2022). 10.1002/smll.202200007 35289495

[24] N. Paracini, L. A. Clifton, M. W. A. Skoda, J. H. Lakey, Liquid crystalline bacterial outer membranes are critical for antibiotic susceptibility. Proc. Natl. Acad. Sci. U.S.A. 115, E7587–E7594 (2018). 30037998 10.1073/pnas.1803975115 PMC6094139

[25] V. Corradi, B. I. Sejdiu, H. Mesa-Galloso, H. Abdizadeh, S. Y. Noskov, S. J. Marrink, D. P. Tieleman, Emerging diversity in lipid-protein interactions. Chem. Rev. 119, 5775–5848 (2019). 30758191 10.1021/acs.chemrev.8b00451 PMC6509647

[26] W. Im, S. Khalid, Molecular simulations of Gram-negative bacterial membranes come of age. Annu. Rev. Phys. Chem. 71, 171–188 (2020). 32070216 10.1146/annurev-physchem-103019-033434

[27] P. Rassam, N. A. Copeland, O. Birkholz, C. Tóth, M. Chavent, A. L. Duncan, S. J. Cross, N. G. Housden, R. Kaminska, U. Seger, D. M. Quinn, T. J. Garrod, M. S. P. Sansom, J. Piehler, C. G. Baumann, C. Kleanthous, Supramolecular assemblies underpin turnover of outer membrane proteins in bacteria. Nature 523, 333–336 (2015). 26061769 10.1038/nature14461 PMC4905513

[28] T. S. Ursell, E. H. Trepagnier, K. C. Huang, J. A. Theriot, Analysis of surface protein expression reveals the growth pattern of the Gram-negative outer membrane. PLOS Comput. Biol. 8, e1002680 (2012). 23028278 10.1371/journal.pcbi.1002680 PMC3459847

[29] G. Mamou, F. Corona, R. Cohen-Khait, N. G. Housden, V. Yeung, D. Sun, P. Sridhar, M. Pazos, T. J. Knowles, C. Kleanthous, W. Vollmer, Peptidoglycan maturation controls outer membrane protein assembly. Nature 606, 953–959 (2022). 35705811 10.1038/s41586-022-04834-7 PMC9242858

[30] G. Benn, I. V. Mikheyeva, P. G. Inns, J. C. Forster, N. Ojkic, C. Bortolini, M. G. Ryadnov, C. Kleanthous, T. J. Silhavy, B. W. Hoogenboom, Phase separation in the outer membrane of *Escherichia coli*. Proc. Natl. Acad. Sci. U.S.A. 118, e2112237118 (2021). 34716276 10.1073/pnas.2112237118 PMC8612244

[31] J. Vergalli, I. V. Bodrenko, M. Masi, L. Moynié, S. Acosta-Gutiérrez, J. H. Naismith, A. Davin-Regli, M. Ceccarelli, B. van den Berg, M. Winterhalter, J. M. Pagès, Porins and small-molecule translocation across the outer membrane of Gram-negative bacteria. Nat. Rev. Microbiol. 18, 164–176 (2020). 31792365 10.1038/s41579-019-0294-2

[32] G. W. Li, D. Burkhardt, C. Gross, J. S. Weissman, Quantifying absolute protein synthesis rates reveals principles underlying allocation of cellular resources. Cell 157, 624–635 (2014). 24766808 10.1016/j.cell.2014.02.033 PMC4006352

[33] K. B. Jansen, P. G. Inns, N. G. Housden, J. T. S. Hopper, R. Kaminska, S. Lee, C. V. Robinson, H. Bayley, C. Kleanthous, Bifurcated binding of the OmpF receptor underpins import of the bacteriocin colicin N into *Escherichia coli*. J. Biol. Chem. 295, 9147–9156 (2020). 32398259 10.1074/jbc.RA120.013508 PMC7335789

[34] P. Rassam, K. R. Long, R. Kaminska, D. J. Williams, G. Papadakos, C. G. Baumann, C. Kleanthous, Intermembrane crosstalk drives inner-membrane protein organization in *Escherichia coli*. Nat. Commun. 9, 1082 (2018). 29540681 10.1038/s41467-018-03521-4 PMC5852019

[35] A. Six, K. Mosbahi, M. Barge, C. Kleanthous, T. Evans, D. Walker, Pyocin efficacy in a murine model of *Pseudomonas aeruginosa* sepsis. J. Antimicrob. Chemother. 76, 2317–2324 (2021). 34142136 10.1093/jac/dkab199 PMC8361349

[36] M. Chavent, A. L. Duncan, P. Rassam, O. Birkholz, J. Hélie, T. Reddy, D. Beliaev, B. Hambly, J. Piehler, C. Kleanthous, M. S. P. Sansom, How nanoscale protein interactions determine the mesoscale dynamic organisation of bacterial outer membrane proteins. Nat. Commun. 9, 2846 (2018). 30030429 10.1038/s41467-018-05255-9 PMC6054660

[37] M. Chavent, A. L. Duncan, M. S. P. Sansom, Molecular dynamics simulations of membrane proteins and their interactions: From nanoscale to mesoscale. Curr. Opin. Struct. Biol. 40, 8–16 (2016). 27341016 10.1016/j.sbi.2016.06.007 PMC5404110

[38] D. S. Patel, Y. Qi, W. Im, Modeling and simulation of bacterial outer membranes and interactions with membrane proteins. Curr. Opin. Struct. Biol. 43, 131–140 (2017). 28157627 10.1016/j.sbi.2017.01.003

[39] W. Arunmanee, M. Pathania, A. S. Solovyova, A. P. le Brun, H. Ridley, A. Baslé, B. van den Berg, J. H. Lakey, Gram-negative trimeric porins have specific LPS binding sites that are essential for porin biogenesis. Proc. Natl. Acad. Sci. U.S.A. 113, E5034–E5043 (2016). 27493217 10.1073/pnas.1602382113 PMC5003275

[40] J. Shearer, D. Jefferies, S. Khalid, Outer membrane proteins OmpA, FhuA, OmpF, EstA, BtuB, and OmpX have unique lipopolysaccharide fingerprints. J. Chem. Theory Comput. 15, 2608–2619 (2019). 30848905 10.1021/acs.jctc.8b01059

[41] A. D. Ferguson, W. Welte, E. Hofmann, B. Lindner, O. Holst, J. W. Coulton, K. Diederichs, A conserved structural motif for lipopolysaccharide recognition by procaryotic and eucaryotic proteins. Structure 8, 585–592 (2000). 10873859 10.1016/s0969-2126(00)00143-x

[42] N. G. Housden, J. T. S. Hopper, N. Lukoyanova, D. Rodriguez-Larrea, J. A. Wojdyla, A. Klein, R. Kaminska, H. Bayley, H. R. Saibil, C. V. Robinson, C. Kleanthous, Intrinsically disordered protein threads through the bacterial outer-membrane porin OmpF. Science 340, 1570–1574 (2013). 23812713 10.1126/science.1237864 PMC3856478

[43] Y. Kamio, H. Nikaido, Outer membrane of *Salmonella typhimurium*: Accessibility of phospholipid head groups to phospholipase c and cyanogen bromide activated dextran in the external medium. Biochemistry 15, 2561–2570 (1976). 820368 10.1021/bi00657a012

[44] K. B. Gehring, H. Nikaido, Existence and purification of porin heterotrimers of *Escherichia coli* K12 OmpC, OmpF, and PhoE proteins. J. Biol. Chem. 264, 2810–2815 (1989). 2464593

[45] S. Ichihara, S. Mizushima, Arrangement of proteins O-8 and O-9 in outer membrane of *Escherichia coli* K-12: Existence of homotrimers and heterotrimers. Eur. J. Biochem. 100, 321–328 (1979). 389623 10.1111/j.1432-1033.1979.tb04174.x

[46] I. Wittig, H.-P. Braun, H. Schägger, Blue native PAGE. Nat. Protoc. 1, 418–428 (2006). 17406264 10.1038/nprot.2006.62

[47] F. A. Schabert, C. Henn, A. Engel, Native *Escherichia coli* OmpF porin surfaces probed by atomic force microscopy. Science 268, 92–94 (1995). 7701347 10.1126/science.7701347

[48] I. Casuso, J. Khao, M. Chami, P. Paul-Gilloteaux, M. Husain, J. P. Duneau, H. Stahlberg, J. N. Sturgis, S. Scheuring, Characterization of the motion of membrane proteins using high-speed atomic force microscopy. Nat. Nanotechnol. 7, 525–529 (2012). 22772862 10.1038/nnano.2012.109

[49] S. Jarosławski, K. Duquesne, J. N. Sturgis, S. Scheuring, High-resolution architecture of the outer membrane of the Gram-negative bacteria *Roseobacter denitrificans*. Mol. Microbiol. 74, 1211–1222 (2009). 19843216 10.1111/j.1365-2958.2009.06926.x

[50] H. Yamashita, A. Taoka, T. Uchihashi, T. Asano, T. Ando, Y. Fukumori, Single-molecule imaging on living bacterial cell surface by high-speed AFM. J. Mol. Biol. 422, 300–309 (2012). 22613761 10.1016/j.jmb.2012.05.018

[51] S. Khalid, T. J. Piggot, F. Samsudin, Atomistic and coarse grain simulations of the cell envelope of Gram-negative bacteria: What have we learned? Acc. Chem. Res. 52, 180–188 (2019). 30562009 10.1021/acs.accounts.8b00377

[52] S. D. Gunasinghe, T. Shiota, C. J. Stubenrauch, K. E. Schulze, C. T. Webb, A. J. Fulcher, R. A. Dunstan, I. D. Hay, T. Naderer, D. R. Whelan, T. D. M. Bell, K. D. Elgass, R. A. Strugnell, T. Lithgow, The WD40 protein BamB mediates coupling of BAM complexes into assembly precincts in the bacterial outer membrane. Cell Rep. 23, 2782–2794 (2018). 29847806 10.1016/j.celrep.2018.04.093

[53] J. Szczepaniak, P. Holmes, K. Rajasekar, R. Kaminska, F. Samsudin, P. G. Inns, P. Rassam, S. Khalid, S. M. Murray, C. Redfield, C. Kleanthous, The lipoprotein Pal stabilises the bacterial outer membrane during constriction by a mobilisation-and-capture mechanism. Nat. Commun. 11, 1305 (2020). 32161270 10.1038/s41467-020-15083-5 PMC7066135

[54] C. Kleanthous, P. Rassam, C. G. Baumann, Protein-protein interactions and the spatiotemporal dynamics of bacterial outer membrane proteins. Curr. Opin. Struct. Biol. 35, 109–115 (2015). 26629934 10.1016/j.sbi.2015.10.007 PMC4684144

[55] P.-C. Hsu, F. Samsudin, J. Shearer, S. Khalid, It is complicated: Curvature, diffusion, and lipid sorting within the two membranes of *Escherichia coli*. J. Phys. Chem. Lett. 8, 5513–5518 (2017). 29053278 10.1021/acs.jpclett.7b02432

[56] S. Alvira, D. W. Watkins, L. Troman, W. J. Allen, J. S. Lorriman, G. Degliesposti, E. J. Cohen, M. Beeby, B. Daum, V. A. M. Gold, J. M. Skehel, I. Collinson, Inter-membrane association of the Sec and BAM translocons for bacterial outer-membrane biogenesis. eLife 9, e60669 (2020). 33146611 10.7554/eLife.60669 PMC7695460

[57] E. R. Rojas, G. Billings, P. D. Odermatt, G. K. Auer, L. Zhu, A. Miguel, F. Chang, D. B. Weibel, J. A. Theriot, K. C. Huang, The outer membrane is an essential load-bearing element in Gram-negative bacteria. Nature 559, 617–621 (2018). 30022160 10.1038/s41586-018-0344-3 PMC6089221

[58] J. Sun, S. T. Rutherford, T. J. Silhavy, K. C. Huang, Physical properties of the bacterial outer membrane. Nat. Rev. Microbiol. 20, 236–248 (2022). 34732874 10.1038/s41579-021-00638-0 PMC8934262

[59] M. T. Doyle, J. R. Jimah, T. Dowdy, S. I. Ohlemacher, M. Larion, J. E. Hinshaw, H. D. Bernstein, Cryo-EM structures reveal multiple stages of bacterial outer membrane protein folding. Cell 185, 1143–1156.e13 (2022). 35294859 10.1016/j.cell.2022.02.016 PMC8985213

[60] Z.-S. Chong, W.-F. Woo, S.-S. Chng, Osmoporin OmpC forms a complex with MlaA to maintain outer membrane lipid asymmetry in *Escherichia coli*. Mol. Microbiol. 98, 1133–1146 (2015). 26314242 10.1111/mmi.13202

[61] J. Abellón-Ruiz, S. S. Kaptan, A. Baslé, B. Claudi, D. Bumann, U. Kleinekathöfer, B. van den Berg, Structural basis for maintenance of bacterial outer membrane lipid asymmetry. Nat. Microbiol. 2, 1616–1623 (2017). 29038444 10.1038/s41564-017-0046-x

[62] M. Z. Islam, A. Fokine, M. Mahalingam, Z. Zhang, C. Garcia-Doval, M. J. van Raaij, M. G. Rossmann, V. B. Rao, Molecular anatomy of the receptor binding module of a bacteriophage long tail fiber. PLOS Pathog. 15, e1008193 (2019). 31856258 10.1371/journal.ppat.1008193 PMC6957217

[63] A. von Kügelgen, H. Tang, G. G. Hardy, D. Kureisaite-Ciziene, Y. V. Brun, P. J. Stansfeld, C. V. Robinson, T. A. M. Bharat, *In situ* structure of an intact lipopolysaccharide-bound bacterial surface layer. Cell 180, 348–358.e15 (2020). 31883796 10.1016/j.cell.2019.12.006 PMC6978808

[64] T. A. M. Bharat, A. von Kügelgen, V. Alva, Molecular logic of prokaryotic surface layer structures. Trends Microbiol. 29, 405–415 (2021). 33121898 10.1016/j.tim.2020.09.009 PMC8559796

[65] N. G. Housden, S. R. Loftus, G. R. Moore, R. James, C. Kleanthous, Cell entry mechanism of enzymatic bacterial colicins: Porin recruitment and the thermodynamics of receptor binding. Proc. Natl. Acad. Sci. U.S.A. 102, 13849–13854 (2005). 16166265 10.1073/pnas.0503567102 PMC1236540

[66] R. Cohen-Khait, A. Harmalkar, P. Pham, M. N. Webby, N. G. Housden, E. Elliston, J. T. S. Hopper, S. Mohammed, C. V. Robinson, J. J. Gray, C. Kleanthous, Colicin-mediated transport of DNA through the iron transporter FepA. MBio 12, e0178721 (2021). 34544275 10.1128/mBio.01787-21 PMC8546555

[67] M.-L. R. Francis, M. N. Webby, N. G. Housden, R. Kaminska, E. Elliston, B. Chinthammit, N. Lukoyanova, C. Kleanthous, Porin threading drives receptor disengagement and establishes active colicin transport through *Escherichia coli* OmpF. EMBO J. 40, e108610 (2021). 34515361 10.15252/embj.2021108610 PMC8561637

[68] K. M. Storek, J. Chan, R. Vij, N. Chiang, Z. Lin, J. Bevers III, C. M. Koth, J.-M. Vernes, Y. G. Meng, J. Yin, H. Wallweber, O. Dalmas, S. Shriver, C. Tam, K. Schneider, D. Seshasayee, G. Nakamura, P. A. Smith, J. Payandeh, J. T. Koerber, L. Comps-Agrar, S. T. Rutherford, Massive antibody discovery used to probe structure-function relationships of the essential outer membrane protein LptD. eLife 8, e46258 (2019). 31237236 10.7554/eLife.46258 PMC6592684

[69] G. Klein, B. Lindner, H. Brade, S. Raina, Molecular basis of lipopolysaccharide heterogeneity in *Escherichia coli*: Envelope stress-responsive regulators control the incorporation of glycoforms with a third 3-deoxy-α-D-manno-oct-2-ulosonic acid and rhamnose. J. Biol. Chem. 286, 42787–42807 (2011). 22021036 10.1074/jbc.M111.291799 PMC3234843

[70] A. Washizaki, T. Yonesaki, Y. Otsuka, Characterization of the interactions between *Escherichia coli* receptors, LPS and OmpC, and bacteriophage T4 long tail fibers. MicrobiologyOpen 5, 1003–1015 (2016). 27273222 10.1002/mbo3.384 PMC5221442

[71] D. Liu, P. R. Reeves, *Escherichia coli* K12 regains its O antigen. Microbiology 140, 49–57 (1994). 7512872 10.1099/13500872-140-1-49

[72] D. F. Browning, T. J. Wells, F. L. S. França, F. C. Morris, Y. R. Sevastsyanovich, J. A. Bryant, M. D. Johnson, P. A. Lund, A. F. Cunningham, J. L. Hobman, R. C. May, M. A. Webber, I. R. Henderson, Laboratory adapted *Escherichia coli* K-12 becomes a pathogen of *Caenorhabditis elegans* upon restoration of O antigen biosynthesis. Mol. Microbiol. 87, 939–950 (2013). 23350972 10.1111/mmi.12144

[73] P. White, A. Joshi, P. Rassam, N. G. Housden, R. Kaminska, J. D. Goult, C. Redfield, L. C. McCaughey, D. Walker, S. Mohammed, C. Kleanthous, Exploitation of an iron transporter for bacterial protein antibiotic import. Proc. Natl. Acad. Sci. U.S.A. 114, 12051–12056 (2017). 29078392 10.1073/pnas.1713741114 PMC5692591

[74] J. Cox, M. Mann, MaxQuant enables high peptide identification rates, individualized p.p.b.-range mass accuracies and proteome-wide protein quantification. Nat. Biotechnol. 26, 1367–1372 (2008). 19029910 10.1038/nbt.1511

[75] S. Purcell, B. Neale, K. Todd-Brown, L. Thomas, M. A. R. Ferreira, D. Bender, J. Maller, P. Sklar, P. I. W. de Bakker, M. J. Daly, P. C. Sham, PLINK: A tool set for whole-genome association and population-based linkage analyses. Am. J. Hum. Genet. 81, 559–575 (2007). 17701901 10.1086/519795 PMC1950838

[76] J. Gault, J. A. C. Donlan, I. Liko, J. T. S. Hopper, K. Gupta, N. G. Housden, W. B. Struwe, M. T. Marty, T. Mize, C. Bechara, Y. Zhu, B. Wu, C. Kleanthous, M. Belov, E. Damoc, A. Makarov, C. V. Robinson, High-resolution mass spectrometry of small molecules bound to membrane proteins. Nat. Methods 13, 333–336 (2016). 26901650 10.1038/nmeth.3771 PMC4856209

[77] G. Young, N. Hundt, D. Cole, A. Fineberg, J. Andrecka, A. Tyler, A. Olerinyova, A. Ansari, E. G. Marklund, M. P. Collier, S. A. Chandler, O. Tkachenko, J. Allen, M. Crispin, N. Billington, Y. Takagi, J. R. Sellers, C. Eichmann, P. Selenko, L. Frey, R. Riek, M. R. Galpin, W. B. Struwe, J. L. P. Benesch, P. Kukura, Quantitative mass imaging of single biological macromolecules. Science 360, 423–427 (2018). 29700264 10.1126/science.aar5839 PMC6103225

[78] G. Loy, A. Zelinsky, Fast radial symmetry for detecting points of interest. IEEE Trans. Pattern Anal. Mach. Intell. 25, 959–973 (2003).

[79] G. Benn, A. L. B. Pyne, M. G. Ryadnov, B. W. Hoogenboom, Imaging live bacteria at the nanoscale: Comparison of immobilisation strategies. Analyst 144, 6944–6952 (2019). 31620716 10.1039/c9an01185d PMC7138128

[80] J. Schindelin, I. Arganda-Carreras, E. Frise, V. Kaynig, M. Longair, T. Pietzsch, S. Preibisch, C. Rueden, S. Saalfeld, B. Schmid, J. Y. Tinevez, D. J. White, V. Hartenstein, K. Eliceiri, P. Tomancak, A. Cardona, Fiji: An open-source platform for biological-image analysis. Nat. Methods 9, 676–682 (2012). 22743772 10.1038/nmeth.2019 PMC3855844

[81] D. Nečas, P. Klapetek, Gwyddion: An open-source software for SPM data analysis. Centr. Eur. J. Phys. 10, 181–188 (2012).

[82] R. G. Efremov, L. A. Sazanov, Structure of *Escherichia coli* OmpF porin from lipidic mesophase. J. Struct. Biol. 178, 311–318 (2012). 22484237 10.1016/j.jsb.2012.03.005

[83] D. M. Freed, P. S. Horanyi, M. C. Wiener, D. S. Cafiso, Conformational exchange in a membrane transport protein is altered in protein crystals. Biophys. J. 99, 1604–1610 (2010). 20816073 10.1016/j.bpj.2010.06.026 PMC2931748

[84] S. K. Buchanan, B. S. Smith, L. Venkatramani, D. Xia, L. Esser, M. Palnitkar, R. Chakraborty, D. van der Helm, J. Deisenhofer, Crystal structure of the outer membrane active transporter FepA from *Escherichia coli*. Nat. Struct. Biol. 6, 56–63 (1999). 9886293 10.1038/4931

[85] R. Grinter, T. Lithgow, Determination of the molecular basis for coprogen import by Gram-negative bacteria. IUCrJ 6, 401–411 (2019). 10.1107/S2052252519002926 PMC6503915 31098021

[86] L. Han, J. Zheng, Y. Wang, X. Yang, Y. Liu, C. Sun, B. Cao, H. Zhou, D. Ni, J. Lou, Y. Zhao, Y. Huang, Structure of the BAM complex and its implications for biogenesis of outer-membrane proteins. Nat. Struct. Mol. Biol. 23, 192–196 (2016). 26900875 10.1038/nsmb.3181

[87] P. C. Hsu, B. M. H. Bruininks, D. Jefferies, P. Cesar Telles de Souza, J. Lee, D. S. Patel, S. J. Marrink, Y. Qi, S. Khalid, W. Im, CHARMM-GUI Martini Maker for modeling and simulation of complex bacterial membranes with lipopolysaccharides. J. Comput. Chem. 38, 2354–2363 (2017). 28776689 10.1002/jcc.24895 PMC5939954

[88] S. Jo, T. Kim, V. G. Iyer, W. Im, CHARMM-GUI: A web-based graphical user interface for CHARMM. J. Comput. Chem. 29, 1859–1865 (2008). 18351591 10.1002/jcc.20945

[89] J. Lee, X. Cheng, J. M. Swails, M. S. Yeom, P. K. Eastman, J. A. Lemkul, S. Wei, J. Buckner, J. C. Jeong, Y. Qi, S. Jo, V. S. Pande, D. A. Case, C. L. Brooks III, A. D. MacKerell Jr., J. B. Klauda, W. Im, CHARMM-GUI input generator for NAMD, GROMACS, AMBER, OpenMM, and CHARMM/OpenMM simulations using the CHARMM36 additive force field. J. Chem. Theory Comput. 12, 405–413 (2016). 26631602 10.1021/acs.jctc.5b00935 PMC4712441

[90] T. A. Wassenaar, H. I. Ingólfsson, R. A. Böckmann, D. P. Tieleman, S. J. Marrink, Computational lipidomics with insane: A versatile tool for generating custom membranes for molecular simulations. J. Chem. Chem. Theory Comput. 11, 2144–2155 (2015). 10.1021/acs.jctc.5b00209 26574417

[91] S. Khalid, C. Schroeder, P. J. Bond, A. L. Duncan, What have molecular simulations contributed to understanding of Gram-negative bacterial cell envelopes? Microbiology (Reading) 168, 001165 (2022). 35294337 10.1099/mic.0.001165 PMC9558347

[92] M. J. Abraham, T. Murtola, R. Schulz, S. Páll, J. C. Smith, B. Hess, E. Lindahl, GROMACS: High performance molecular simulations through multi-level parallelism from laptops to supercomputers. SoftwareX 1-2, 19–25 (2015).

[93] D. H. de Jong, G. Singh, W. F. D. Bennett, C. Arnarez, T. A. Wassenaar, L. V. Schäfer, X. Periole, D. P. Tieleman, S. J. Marrink, Improved parameters for the martini coarse-grained protein force field. J. Chem. Theory Comput. 9, 687–697 (2013). 26589065 10.1021/ct300646g

[94] G. Bussi, D. Donadio, M. Parrinello, Canonical sampling through velocity rescaling. J. Chem. Phys. 126, 014101 (2007). 17212484 10.1063/1.2408420

[95] M. Parrinello, A. Rahman, Polymorphic transitions in single crystals: A new molecular dynamics method. J. Appl. Phys. 52, 7182–7190 (1981).

[96] W. Song, R. A. Corey, T. B. Ansell, C. K. Cassidy, M. R. Horrell, A. L. Duncan, P. J. Stansfeld, M. S. P. Sansom, PyLipID: A python package for analysis of protein–lipid interactions from molecular dynamics simulations. J. Chem. Theory Comput. 18, 1188–1201 (2022). 35020380 10.1021/acs.jctc.1c00708 PMC8830038

