# Supplementary material for: Lipids mediate supramolecular outer membrane protein assembly in bacteria
Source: Sci Adv. 2022 Nov 2;8(44):eadc9566. doi: 10.1126/sciadv.adc9566 (PMC9629720; doi:10.1126/sciadv.adc9566)
Supplement: Supplementary file 1 — Figs. S1 to S19 Tables S1 to S5 [file sciadv.adc9566_sm.pdf]

Science Advances
AAAS

Supplementary Materials for

Lipids mediate supramolecular outer membrane protein assembly in bacteria

Melissa N. Webby *et al.*

Corresponding author: Colin Kleanthous, colin.kleanthous@bioch.ox.ac.uk.

Sci. Adv. **8**, eadc9566 (2022)
DOI: 10.1126/sciadv.adc9566

The PDF file includes:

Figs. S1 to S19
Tables S1 to S5
Legend for movie S1

Other Supplementary Material for this manuscript includes the following:

Movie S1

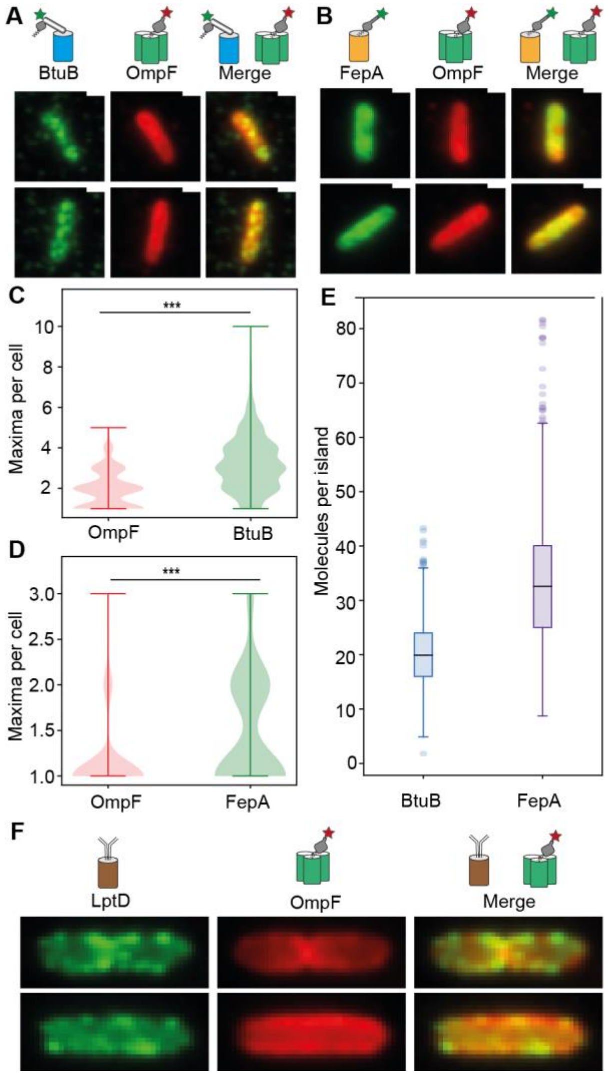

Fig. S1. Clusters of the monomeric OMPs FepA, BtuB and LptD reside within OmpF-rich regions. A, Representative total internal reflection fluorescence microscopy (TIRFM) images (average of 50 frames) of *E. coli* MG1655 cells labelled with ColE9^{AF488} and ColN-mCherry, artificially colored *green* and *red*, binding to BtuB and OmpF, respectively. Images of identical cells from the red and green channels are overlaid to create merged images showing co-labelled OMPs (*yellow*). **B,** Representative TIRFM images (average of 50 frames) of *E. coli* MG1655 cells labelled with ColB-GFP (Binds FepA) and ColN-mCherry, artificially colored *green* and *red*, respectively. Images from the red and green channels are overlaid to create merged image showing

co-labelled OMPs, FepA and OmpF (*yellow*). **C-D,** Local maxima plots from TIRFM images of *E. coli* MG1655 cells labelled with either ColE9^{AF488}, ColB, or ColN-mCherry, for BtuB, FepA and OmpF labelling respectively. Monomeric, low abundance OMPs typically have a higher number of fluorescence maxima per cell suggesting a more localized distribution. Statistical significance was calculated using bootstrapping: data was resampled 100,000 times and the percentile method was used to determine the 95, 99, and 99.9% confidence intervals. Ns= no significant difference, * = $p < 0.05$, ** = $p < 0.01$, *** = $p < 0.001$. **E,** The number of monomeric OMPs per island were estimated from TIRFM images by intensity-based protein counting. Using the bootstrapping method to calculate statistical significance, the number of molecules per island of BtuB and FepA were found to differ significantly at a 99.9% confidence interval. **F,** Epifluorescence images (average of 50 frames) of representative *E. coli* MG1655 cells labelled with AF488 anti-LptD Fab and ColN-mCherry, artificially colored *green* and *red*, respectively. Images from the red and green channels are overlaid to create the merged image, with yellow indicating co-localization of LptD within OmpF regions. Scale bars, 1 µm.

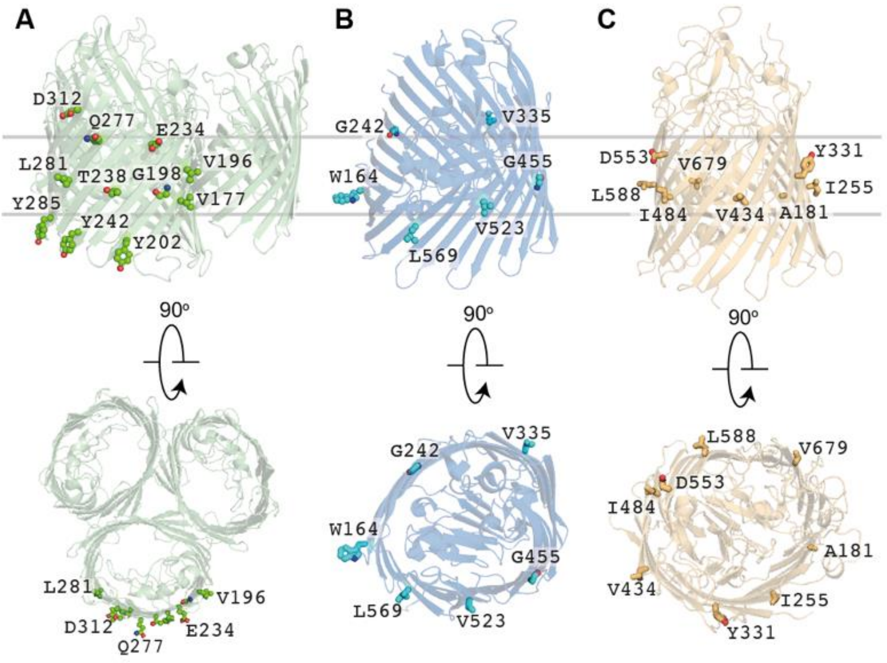

Fig. S2. Distribution of BPA incorporation sites in β-barrel membrane-facing residues of OmpF, BtuB and FepA. A, The individual sites for BPA incorporation in OmpF (PDB ID 3K19) (*85*), represented as sticks, traverse the β-strands of the barrel and are exclusively located on the membrane-facing face of the protein. The 90° rotation highlights the location of the BPA incorporation sites relative to the trimer interface. Sticks are displayed on a single monomer for clarity. Of the active mutants tested (Table S1) only 11/14 are shown, with mid-barrel mutants G211, A251, and I295 excluded for clarity with residue labels. Inactive mutants located closer to the trimer interface are also not shown. **B,** Sites for BPA incorporation in BtuB (PDB ID 1NQF) (*86*), represented as sticks, are predominantly located around the middle of the β-barrel. The 90° rotation highlights the location of the sites, which sample approximately every third strand around the entirety of the barrel. **C,** Sites for BPA incorporation in FepA (PDB ID 1FEP) (*73*), represented as sticks, are predominantly located mid-barrel, with a single mutant ($D553^{BPA}$) located on the extracellular end of a β-strand. The 90° rotation highlights the location of the sites, which sample

the entirety of the barrel. Only variants that were purified and characterized for each OMP are displayed.

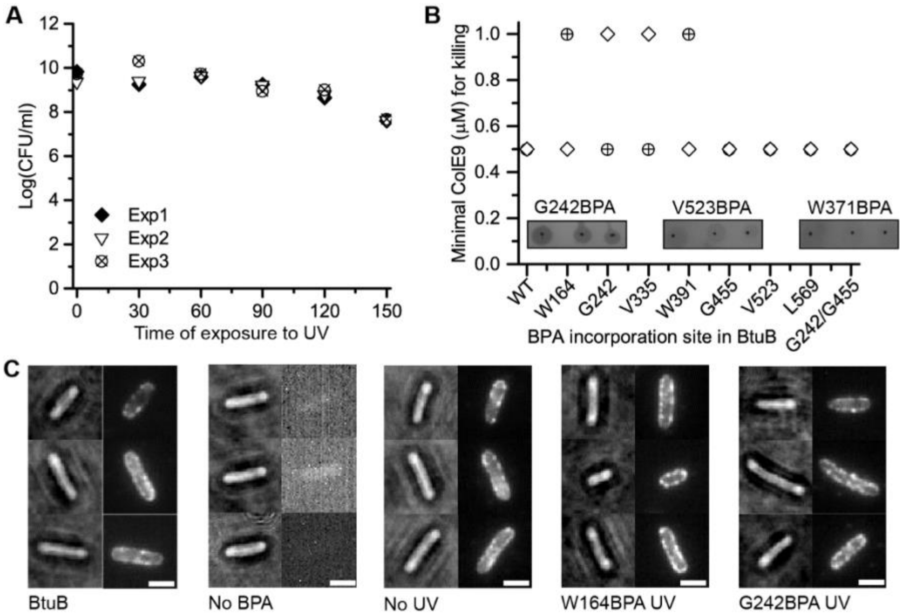

Fig. S3. Functional characterization of BtuB in the *E. coli* OM following site-specific BPA incorporation. A, The viability of *E. coli* RK5016 cells following UV exposure was obtained by determining colony forming units (CFUs) at 30 min intervals up to 150 min. *E. coli* RK5016 cells (50 mL) were grown to an OD_{600} of 0.5 in LB and exposed to UV light (λ_{365}) on ice. 1 mL of sample was removed at each time point, serially diluted, and plated onto LB/agar plates and incubated at 37° C overnight. The resulting colonies were counted and logCFUs plotted against time (n=3 biological repeats). **B,** A colicin-based cytotoxic assay established if $BtuB^{BPA}$ variants were folded and correctly inserted into the OM of *E. coli* RK5016 cells. ColE9 (0.5-10 µM) was spotted onto plates containing *E. coli* RK5016 cells transformed with pEVOL-pBpF and individual $BtuB^{BPA}$ variants expressed from a pBAD vector. The lowest ColE9 concentration where cytotoxicity is observed was plotted for each BPA incorporation site. Biological replicates are displayed as ⊕ and ◊. Representative images of colicin cytotoxicity assays are shown for three mutants $G242^{BPA}$, $V523^{BPA}$ and $W371^{BPA}$. **C,** Representative transillumination and corresponding epifluorescence images (average of 100 frames, 100 ms exposure) of *E. coli* RK5016 cells,

expressing $BtuB^{BPA}$ variants and labelled with $ColE9^{AF647}$. Images show a punctate labelling pattern consistent with the presence of islands. Exposure of cells to UV for 90 min did not appear to alter the distribution of BtuB relative to wild-type cells and no-UV controls. In the absence of BPA, no fluorescence was observed consistent with the lack of BtuB incorporation in the OM. All images are to the same scale. Scale bar, 2 μm. Images are not shown at the same contrast level.

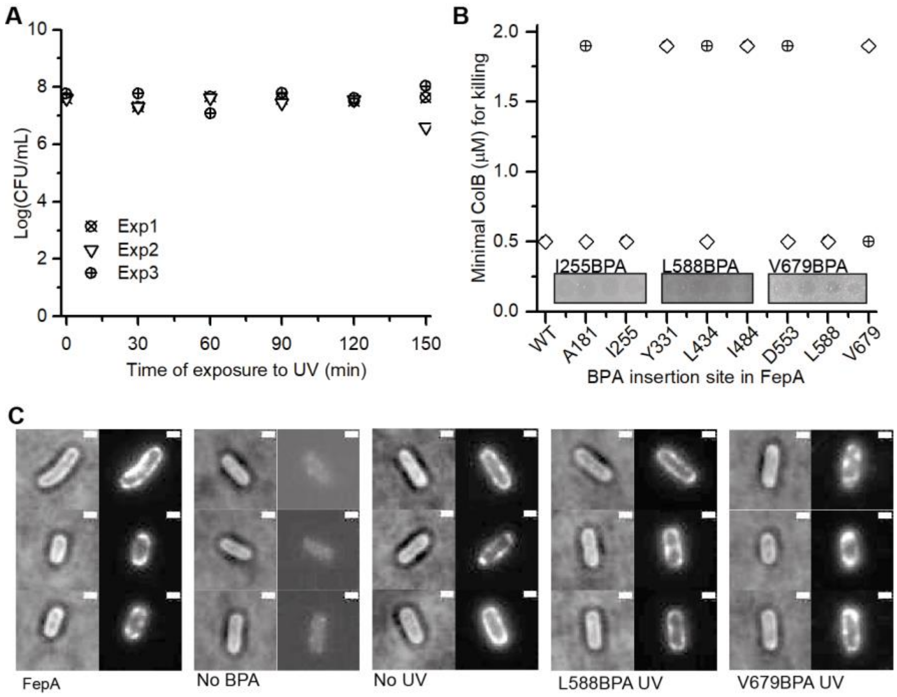

Fig. S4. Functional characterization of FepA in the *E. coli* OM following site-specific BPA incorporation. A, The viability of *E. coli* BW25113 *ΔFepA* cells following UV exposure was obtained by determining CFUs at 30 min intervals up to 150 min. *E. coli* BW25113 cells (50 mL) were grown to OD_{600} of 0.5 and exposed to UV light (λ_{365}) on ice. 1 mL sample was removed at each time point, serially diluted, and plated onto LB/agar plates and incubated at 37° C overnight. The resulting colonies were counted and logCFUs plotted against time (n=3 biological repeats). **B**, A colicin-based cytotoxic assay was used to determine if FepA^{BPA} variants were functional. ColB (0.5-32 µM) was spotted onto plates with *E. coli* BW25113 expressing a specific FepA^{BPA} variant (pBad) and tRNase for BPA incorporation (pEVOL-pBpF plasmid). The lowest ColB concentration where cell killing was observed was plotted for each BPA mutant. Biological replicates are displayed as ⊕ and ◊. Representative images of colicin cytotoxicity plates are shown for three mutants I255^{BPA}, L588^{BPA} and V679^{BPA}. **C**, Transillumination and epifluorescence images (average of 100 frames, 100 ms exposure) of *E. coli* BW25113 cells transformed with the

pEVol-pBpF plasmid and pBAD expression plasmid, encoding individual $FepA^{BPA}$ mutants, were grown to a OD_{600} of 0.6 and protein expression induced with arabinose for 2 h prior to labelling with ColB-GFP. Images show a punctate labelling pattern consistent with the presence of islands. Exposure of cells to UV did not alter the distribution of FepA, when compared to wild-type and the no UV control. In the absence of BPA, no fluorescence was observed, consistent with the lack of FepA in the OM. Scale bar 1 μm. Images are not shown at the same contrast level.

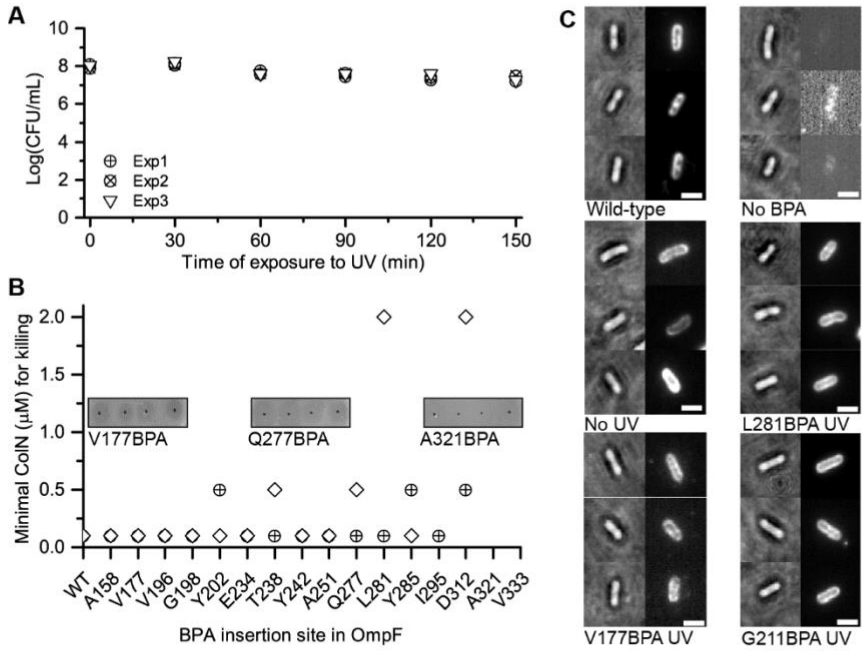

Fig. S5. Functional characterization of OmpF in the *E. coli* OM following site-specific BPA incorporation A, The impact of UV exposure on cell viability for *E. coli* BZB1107 cells was assessed by CFU counts. Cells (50 mL) were grown to OD_{600} 0.5 and exposed to UV light (λ_{365}) on ice. Samples (1 mL) were removed at 30 min intervals, serially diluted, and plated onto LB/agar plates that were incubated at 37 ^{o}C overnight. Resulting colonies were counted and logCFUs plotted against time (n=3 biological repeats). **B,** A colicin-based cytotoxic assay was used to determine if $OmpF^{BPA}$ variants were correctly folded and inserted into the OM of *E. coli* BZB1107 cells transformed with plasmids encoding tRNase for BPA incorporation (pEVOL-pBpF) and $OmpF^{TAG}$ mutant expressed from a pBAD vector. ColN (0.1-8 µM) was spotted onto plates expressing specific $OmpF^{BPA}$ variants. The lowest ColN concentrations where growth clearance was observed are plotted for each BPA incorporation site. Biological replicates are displayed as ⊕ and ◊, for mutants that are resistant to ColN killing such as $A321^{BPA}$ and $V333^{BPA}$, which are located close to the trimer interface, no symbols are shown. Representative images of killing are shown for three mutants $V177^{BPA}$, $Q277^{BPA}$ and $A321^{BPA}$. **C**, Transillumination and

epifluorescence images (average of 100 frames, 100 ms exposure) of *E. coli* BZB1107 cells, transformed with pEVOL-pBpF and pBad plasmids were grown to a OD_{600} of 0.5 and *ompF* expression induced with arabinose for 2 h prior to labelling with ColN-mCherry. Images show OmpF is widely distributed around the periphery of the cells. Exposure of cells to UV for 90 min did not alter the distribution of OmpF appreciably. In the absence of BPA, no fluorescence was observed, consistent with the lack of protein in the membrane. Mutations $A321^{BPA}$ and $V333^{BPA}$ are located close to the trimer interface, suggesting resistance to ColN killing is due to impaired OmpF insertion into the OM. All images are to the same scale. Scale bar, 2 μm. Images are not shown at the same contrast level.

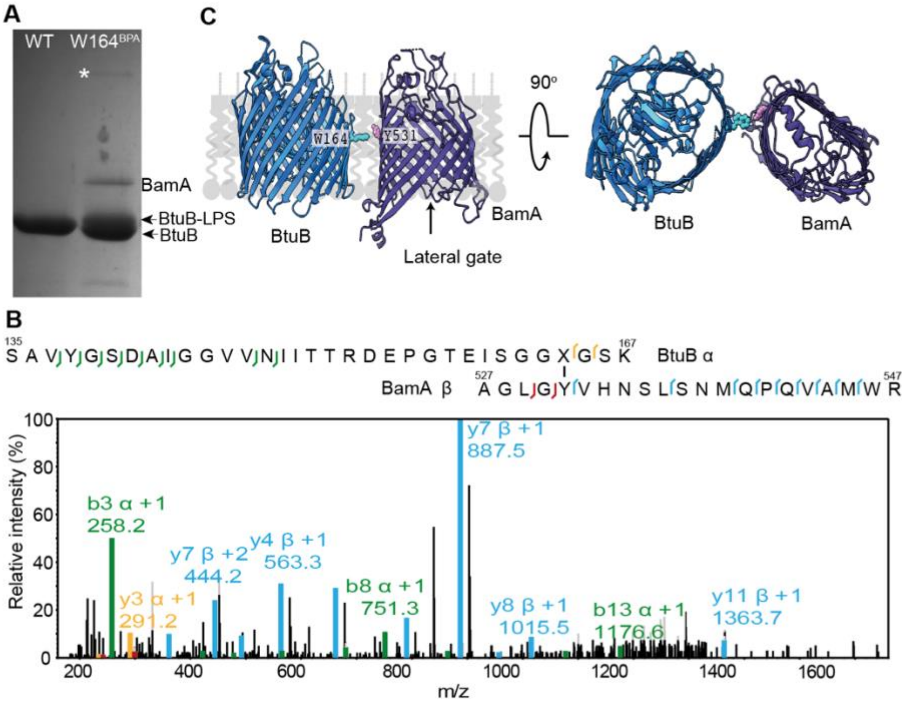

Fig. S6. BtuB and BamA engage in a promiscuous protein-protein interaction in the *E. coli* OM. A, Denaturing SDS-polyacrylamide gel of UV-activated BtuB W164^{BPA} compared to wild-type BtuB. Both proteins were extracted from the OM of *E. coli* RK5016 cells. BtuB W164^{BPA} reproducibly forms an OMP-OMP crosslink, mass ~110 kDa (*white asterisks*). **B**, Fragmentation spectrum for the trypsin-digested 110 kDa crosslinked species in the BtuB W164^{BPA} sample extracted from the SDS gel, shows a direct crosslink to BamA Y531. Crosslinked peptide sequences are shown above. **C**, Crystal structures of BtuB (PDB ID, 1NQF) and BamA (PDB ID, 6FSU) showing the juxtaposition of the crosslinked residues that engage in a promiscuous BtuB-BamA protein-protein interaction (*B*). BamA Y531 is distal to BamA's lateral gate (*87*), between strands 1 and 16, where OMP folding is catalyzed. This interaction likely reflects the dense packing of OMPs, which, in conjunction with their restricted diffusion (*27, 34*), enables a promiscuous protein-protein interaction to form with the OMP biogenesis machinery. Non-crosslinked BamA is also recruited to BtuB via its crosslink to LPS (see main text and Fig. 2A for details).

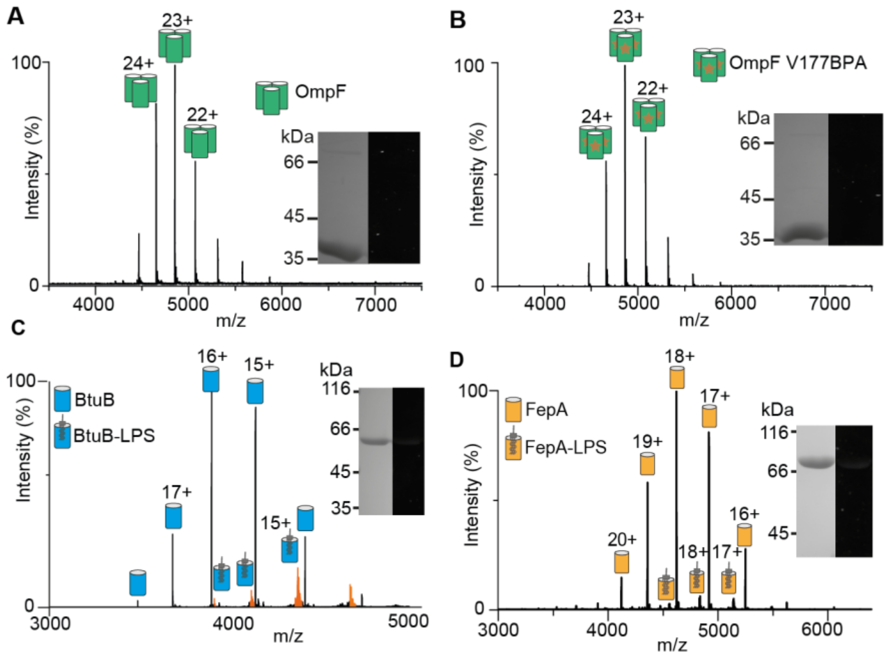

Fig. S7. Native mass spectrometry shows LPS bound to wild-type BtuB and FepA but not OmpF. All protein samples were injected in a buffer containing 1% β-OG and 200 mM ammonium acetate (pH 7.0) at a concentration of 4-50 μM. **A,** Native-MS spectrum for wild-type OmpF released from β-OG micelles. Charge states correspond to an OmpF trimer **B**, Spectrum for OmpF V177^{BPA} without UV irradiation. No bound lipids are detected *Brown star* denotes BPA site. **C**, Native-MS spectrum for wild-type BtuB with peaks corresponding to ligand-free and LPS-bound proteins. **D**, Native-MS spectrum for wild-type FepA with peaks corresponding to ligand-free and LPS-bound proteins. *Inserts*, SDS-polyacrylamide gels showing purified proteins (Coomassie stained, left) and LPS (ProQ emerald green stain, right). No LPS is apparent in any of the samples due to protein denaturation by boiling prior to running the gel. Tabulated native-MS-derived masses for all OMPs are presented in Supplementary Tables S2-S4.

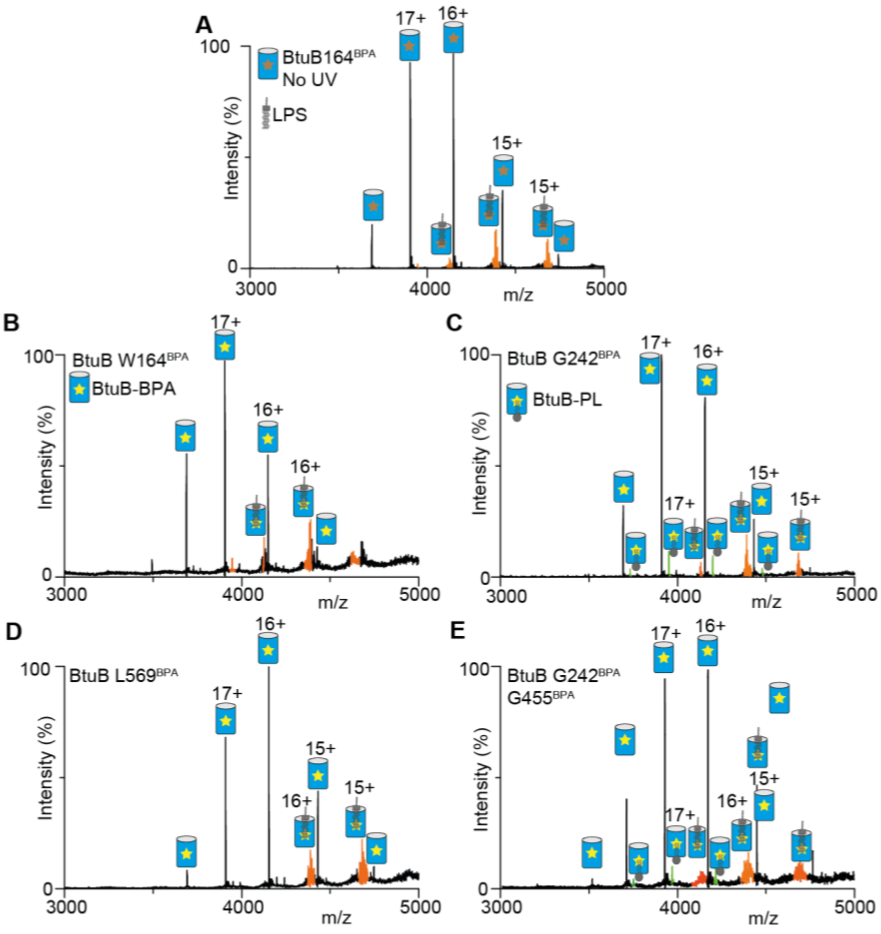

Fig. S8. Assessment of lipid binding to BtuB^{BPA} mutants by native-MS. Shown are the native-MS spectra recorded for BtuB^{BPA} mutants purified from *E. coli* and released from a buffer containing 1% β-OG and 200 mM ammonium acetate (pH 7.0). Peaks corresponding to apo- and LPS-bound proteins are observed in all samples, with phospholipid adducts only observed in samples with the G242^{BPA} mutation. **A,** Native-MS data for BtuB W164^{BPA} purified without UV activation of the BPA crosslinker. Peaks corresponding to ligand-free as well as LPS-bound protein are observed, similar to wild-type BtuB (Supplementary Fig. S7). *Brown* star represents un-activated BPA **B-E**, Native-MS data for BtuB^{BPA} mutants (as labelled in figures) purified after UV activation in 1% β-OG. UV activated BPA is represented as a yellow star, on blue BtuB barrel

with cartoon lipids as appropriate. Tabulated native-MS-derived masses for $BtuB^{BPA}$ variants are presented in Supplementary Table S2.

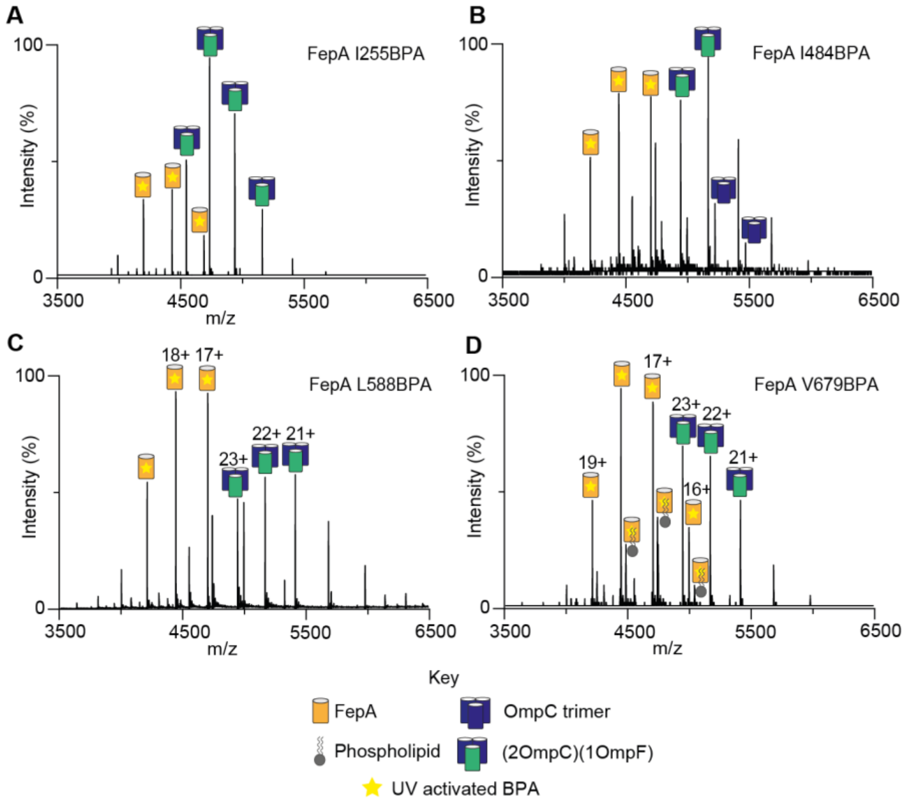

Fig. S9. UV activation of FepA^{BPA} mutants results in the co-purification of porins. All protein samples were injected in a buffer containing 1% β-OG and 200 mM ammonium acetate (pH 7.0) at concentrations ranging from 4-12 μM. **A-D,** Native-MS spectra for four FepA^{BPA} mutants, I255^{BPA} (*panel A*), I484^{BPA} (*panel B*), L588^{BPA} (*panel C*), and V679^{BPA} (*panel D*). Peaks corresponding to apo-FepA (*orange*) were observed in all spectra. In addition, peaks assigned to $(OmpC)_3$ and $(OmpC)_2(OmpF)_1$ were detected in the FepA^{BPA} mutants. The latter proteins are absent in the spectrum for wild-type FepA (Supplementary Fig. S7), suggesting that the photoactivated BPA crosslinker in the FepA mutant promotes association with trimeric OMPs. Complex of phospholipid and the FepA V679^{BPA} mutant was also observed (panel D). LPS was not detected in these spectra, presumably due to their relatively low abundance. Masses are listed in Supplementary Table S3.

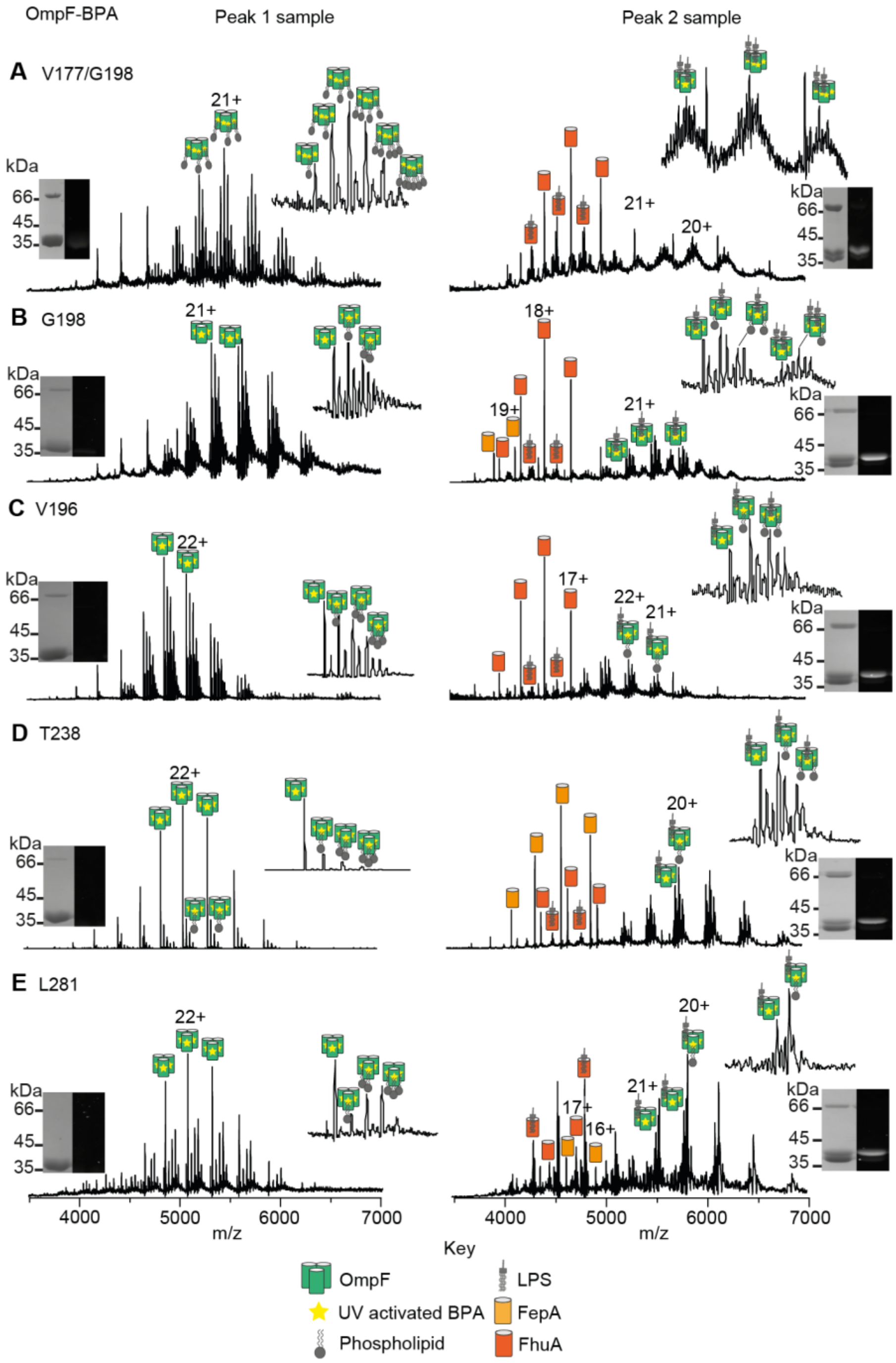

Fig. S10. OmpF^{BPA} mid-barrel mutants crosslink to both LPS and PLs.

Panels are native-MS spectra for OmpF^{BPA} mutants from peak 1 and 2, following UV-activation, membrane extraction, and purification. All samples were injected in a buffer containing 1% β-OG and 200 mM ammonium acetate (pH 7.0). Peak 1 samples are to the left, peak 2 samples to the

right. **A,** OmpF V177^{BPA}/G196^{BPA} double mutant, **B**, OmpF G198^{BPA}, **C**, OmpF V196^{BPA}, **D**, OmpF T238^{BPA}, **E**, OmpF L281^{BPA}. PL-bound OmpF was observed in all peak 1 samples whilst LPS- and PL-bound forms of OmpF were observed in the peak 2 samples. *Insets* show SDS-polyacrylamide gels of each sample analysed that has been stained with Coomassie blue for protein and ProQ emerald green for LPS. In all cases, only peak 2 samples stain for LPS. Together these data show that BPA localized at central locations within the OmpF β-barrel are similarly able to crosslink to PLs or LPS. Spectra for the peak 2 fractions suggest that OmpF crosslinking to LPS facilitates its interactions with the TBDTs FhuA and FepA, which are evident in the native-MS spectra for every mutant. For the V177^{BPA}/G196^{BPA} double mutant, we detect up to 6 PLs, and up to two LPS molecules bound to OmpF trimer. Masses are presented in Supplementary Table S4.

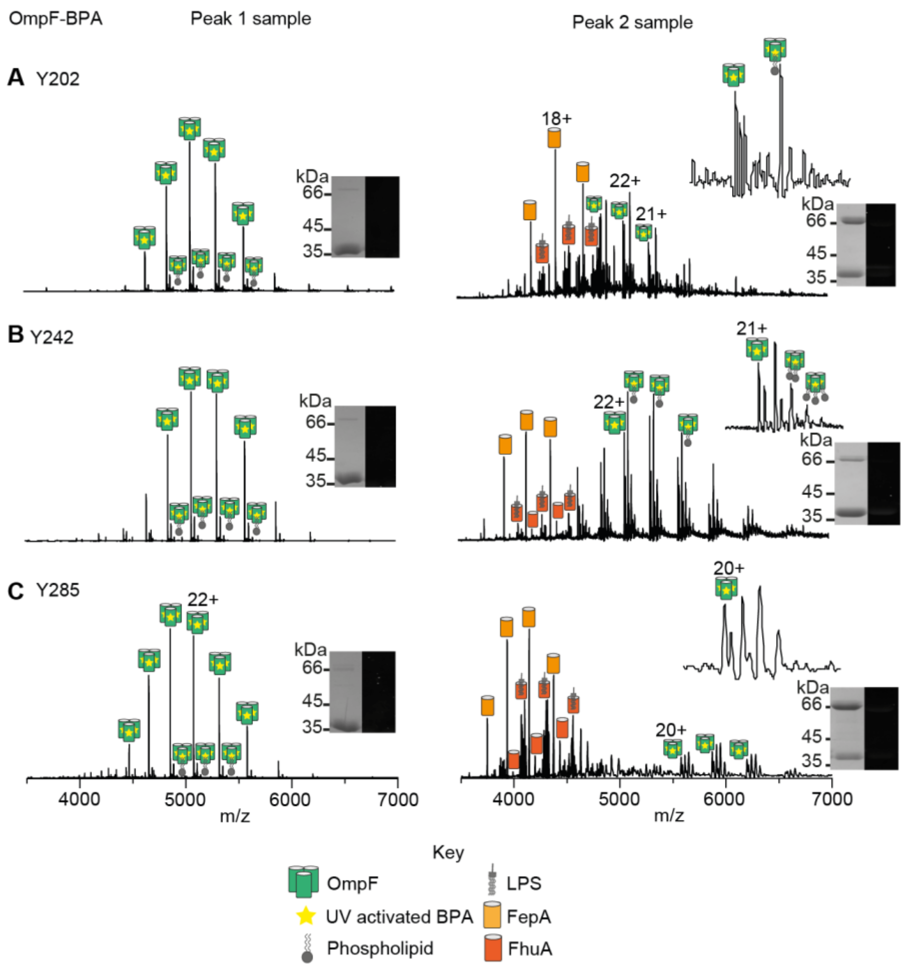

Fig. S11. BPA located towards the periplasmic end of OmpF β-strands only crosslinks to phospholipids. Shown are the native-MS spectra for OmpF^{BPA} mutants Y202^{BPA}, Y264^{BPA}, and Y307^{BPA} purified after UV activation. All samples were injected in a buffer containing 1% β-OG and 200 mM ammonium acetate (pH 7.0). Peaks corresponding to apo-OmpF and PL-bound forms of OmpF, were observed in both peak 1 and peak 2 samples. For the peak 2 fraction of the OmpF Y202^{BPA} mutant, charge states assigned to cardiolipin-bound OmpF were observed. No LPS crosslinks were detected in these mutants, consistent with these aromatic girdle residues interacting more closely with PLs. Here, the peaks assigned to FhuA are more abundant, suggesting that OmpF interactions to FhuA are likely mediated by covalently linked PLs or non-covalent LPS bound to FhuA, as detected in spectra. SDS-PAGE gels of samples stained with Coomassie blue and

emerald lipid stain are shown as insets, confirming the absence of LPS crosslinks in all cases. Tabulated native-MS-derived masses for $OmpF^{BPA}$ variants are presented in Supplementary Table S4.

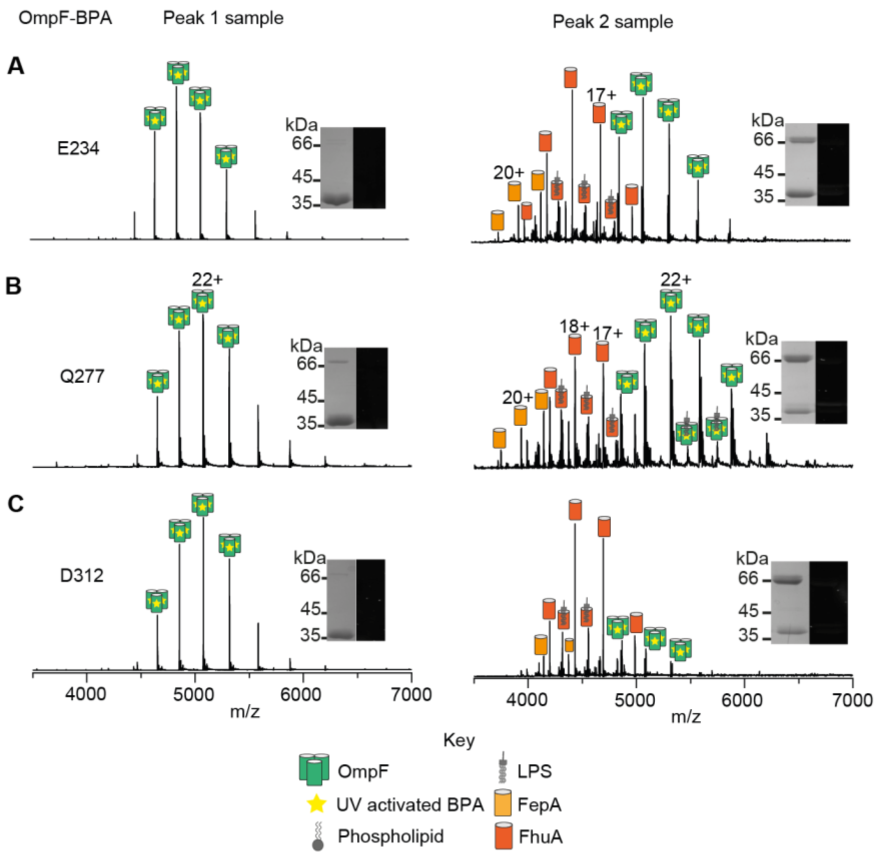

Fig. S12. BPA incorporation at the extracellular end of OmpF β-strands result in limited lipid crosslinks. Native-MS data of OmpF^{BPA} mutants E234^{BPA}, Q277^{BPA}, and D312^{BPA} purified after UV activation. All samples were injected in a buffer containing 1% β-OG and 200 mM ammonium acetate (pH 7.0). For the Q277^{BPA} mutant, ligand-free and LPS-bound OmpF were observed in the peak 2 sample. Whilst the other OmpF^{BPA} mutants were detected mainly in their ligand-free form in peak 1 and 2 samples. Charge states corresponding to FepA, FhuA, and LPS-bound FhuA were observed in the spectrum for the peak 2 fractions of all mutants Although little to no LPS-bound OmpF is detected in these mutants, the presence of co-purified FepA and FhuA suggest that LPS non-covalently bound to monomeric barrels may be mediating these interactions.

SDS-polyacrylamide gels of samples stained with Coomassie blue for protein and ProQ emerald green for LPS are shown as insets.

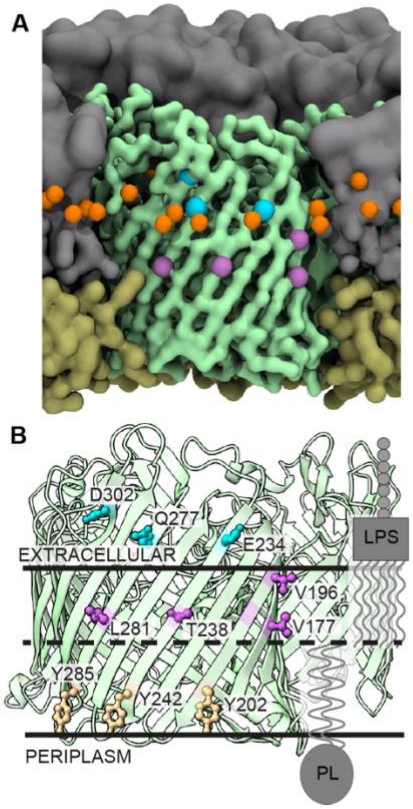

Fig. S13. The patterns of OmpF^{BPA} crosslinks reflect the asymmetry of the *E. coli* outer membrane *in vivo*. A, Simulation data based on BPA crosslinking results, showing the placement of LPS (*grey*) relative to membrane embedded OmpF (*green*). Divalent cations (*orange spheres*) form interactions with the head-group of LPS. In close proximity to these metal ions are OmpF residues located in the upper barrel (*cyan spheres*) that when mutated to BPA, result in few lipid crosslinks due to the limited number of accessible C-H bonds in this region of LPS. Incorporation of BPA into OmpF at mid-barrel sites (*magenta spheres*) result in lipid crosslinks because this region of the protein is surrounded by accessible C-H bonds within the lipid tails of both PLs (*olive*) and LPS. **B,** Schematic of the OmpF trimer (PDB ID, 3K19), with BPA-incorporation sites (*sticks*) represented on a single monomer. Sites that exclusively crosslink to PL in the inner leaflet are shown in *pale orange*, sites that do not form any crosslinks are shown in *cyan*, and sites accessible to both outer and inner leaflet lipids are shown in *magenta*. LPS and PL are illustrated

through cartoons that show the relative position of respective C-H tails, overlapping within the mid-section of OmpF.

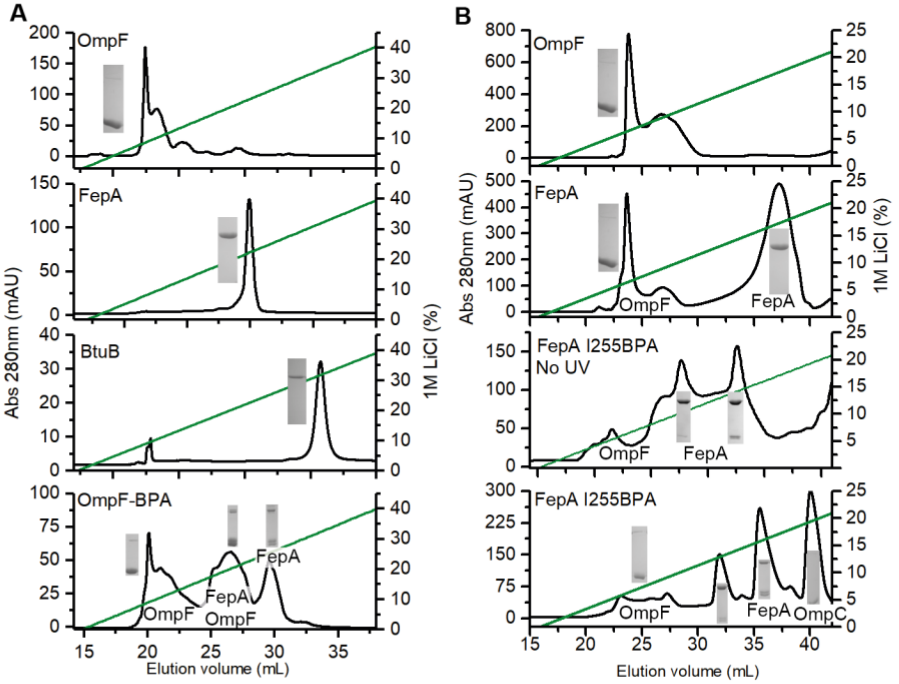

Fig. S14. Separation of target OMPs by ion exchange chromatography. A, Zoom-in of UV (280 nm) recording showing purified OMP (OmpF, FepA, BtuB, UV activated OmpF^{BPA}) elution positions when applying the same LiCl gradient (0-60% 1M LiCl over 14 CV) to an ion exchange column (MonoQ 4.6/100 PE, Cytiva). *Insets* of Coomassie-stained SDS-PAGE gel for each peak are shown. **B,** Zoom-in of UV (280 nm) recording for OmpF and FepA when applying a shallower LiCl gradient (0-60% 1M LiCl over 30 CV) on an ion exchange column (MonoQ 4.6/100 PE, Cytiva). The OmpF sample is the same as that in A. The FepA sample by contrast is a crude sample contaminated with OmpF. The proteins are nevertheless separated by this chromatographic step. Crude FepA I255^{BPA} without UV activation results in two FepA peaks, the second of which contains some OmpC contamination. UV activation of this mutant substantially changes the elution profile; two FepA peaks are resolved as determined by SDS-PAGE. The first peak is largely pure FepA whilst the second peak contains OmpC. OmpC heavily contaminates the sample, eluting towards the end of the run.

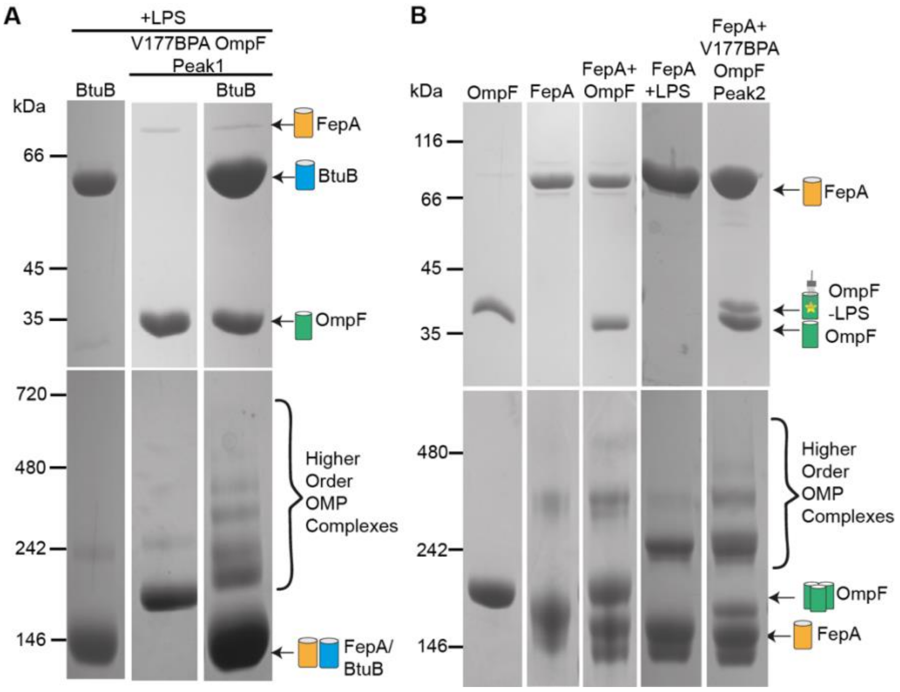

Fig. S15. Promiscuous, non-covalent complexes between OmpF and TBDTs are promoted by LPS. A, The impact of exogenously added LPS on the association of peak 1 OmpF V177^{BPA} (PL crosslinked, accompanied by co-purified FepA) with added BtuB. Sample composition is shown using denaturing SDS-polyacrylamide gels (*top*) while non-covalent assemblies are examined using blue-native PAGE (*bottom*). Addition of purified BtuB (15 µM) to OmpF V177^{BPA} (10 µM) in the presence of exogenous LPS (0.1 mg/mL) resulted in banding in blue native PAGE indicative of the formation of heterologous OMP complexes. Controls of the individual proteins plus LPS exhibit faint bands at high molecular weights suggesting both promiscuous assemblies of OmpF with TBDTs and OMP self-association is occurring. **B,** SDS-PAGE (*top*) and Blue native PAGE (*bottom*) analysis of wild-type OmpF and FepA (20 µM) and mixtures of FepA with wild-type OmpF, exogenous LPS (0.1 mg/mL), or OmpF V177^{BPA} peak 2 (20 µM) suggest that self-association occurs more readily than hetero-association, and LPS (+LPS) amplifies oligomerization. Proteins run as a single band in SDS-PAGE corresponding to the mass of monomeric species. Under native conditions, FepA samples contain two distinct monomer

populations, possibly +/- non-covalent LPS, and dimers, whilst OmpF runs solely as a trimer. The smearing and double banding of FepA makes interpretation of higher molecular weight species ambiguous.

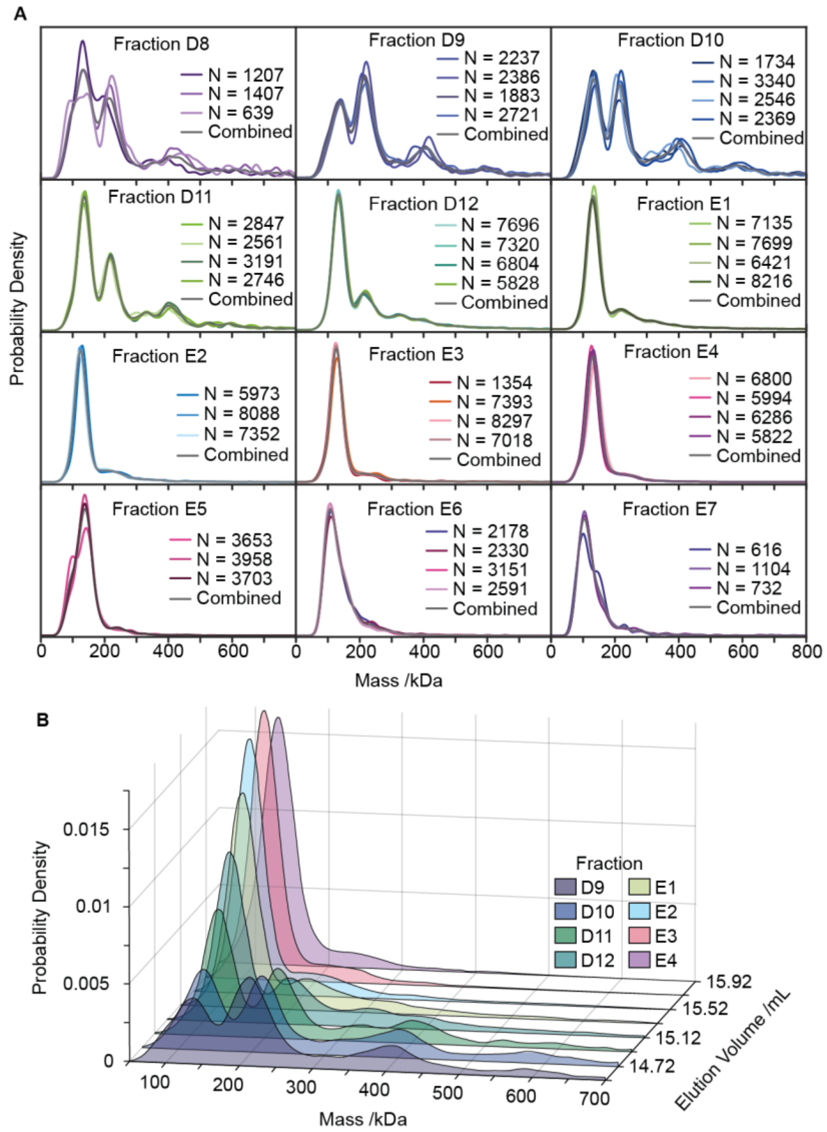

Fig. S16. Heterologous OMP association observed by mass photometry. Purified OMPs were exchanged from a 1% β-OG buffer into amphipol following size exclusion chromatography, to improve image contrast prior to acquisition of mass photometry data. Single particle mass photometry data for gel-filtration fractions are shown. **A,** For each SEC fraction analysed the species analysis plots of mass vs probability density are shown for >3 recordings, with the average of all recordings plotted for comparison. **B,** Species analyses of average plots for each SEC fraction show several high molecular mass species present in samples eluting earlier from the column.

Calculated masses are consistent with both homologous and heterologous OMP complexes in these fractions, including OmpF bound to three BtuB proteins.

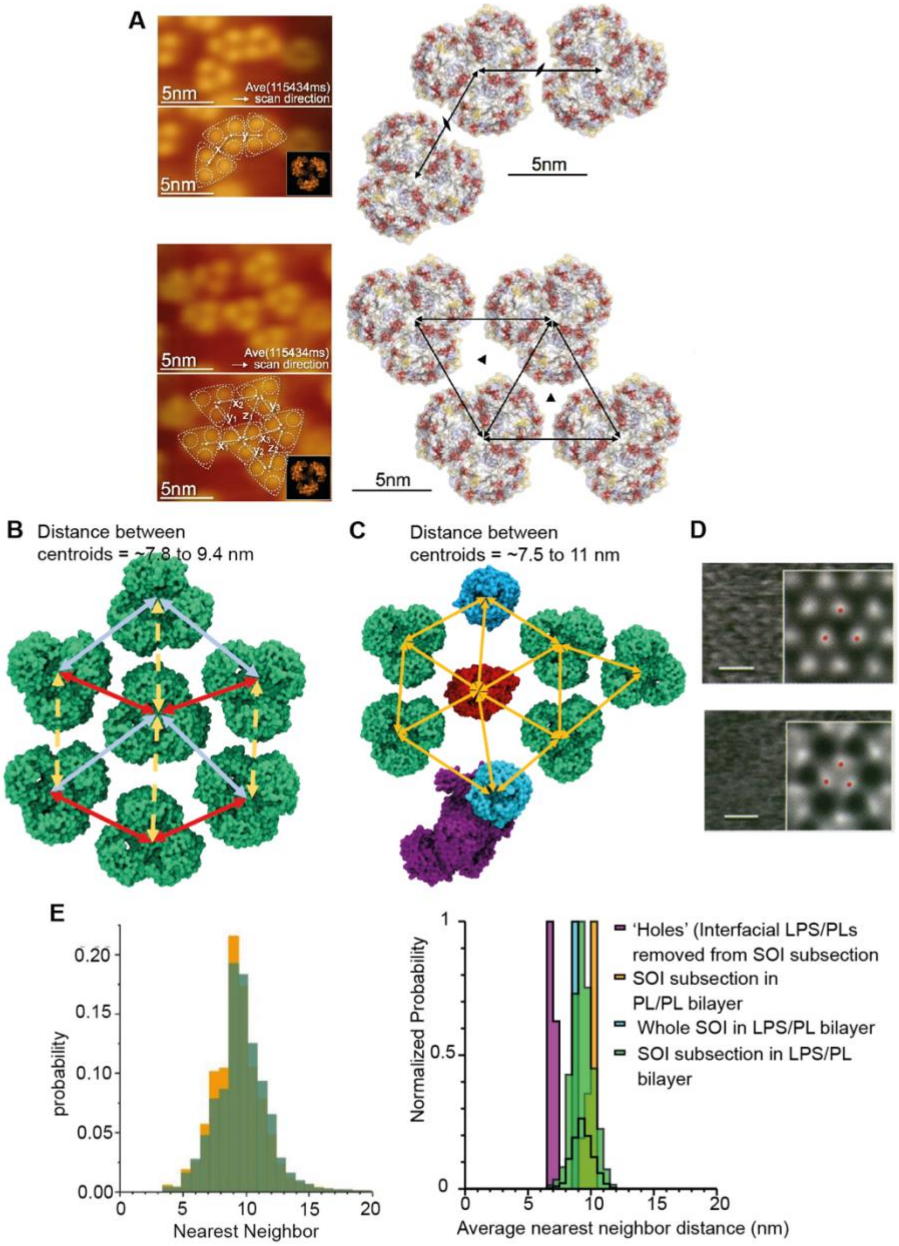

Fig. S17. Building and validating the simulated OMP island. A, Analysis of the most abundant OmpF associations *in vitro* in *E.coli* observed by AFM in supported bilayers. Figure taken, with permission, from Casuso *et al* (*44*). Base-to-base distances estimated here are 7.2 nm, while base-to-tip brings them to 8.6 nm. **B, and C**, Snapshots of OMP clustering from the SOI, which was built around the OmpF clusters observed previously by AFM. **B**, Homologous hexagonal associations among OmpF trimers; centroid distances are ~7.8 and 9.4 nm. The most common

association modes are depicted in the hexagonal arrangement: base-to-base (*solid red arrow*), base-to-tip (*dashed yellow arrow*), and tip-to-tip (*solid blue arrow*). **C**, Heterologous associations of OMPs forming a hexagonal arrangement, with centroid distances ranging from ~7.5 to 11.0 nm. **D**, AFM images of 2D OmpF-DMPC crystals showing two possible conformations of OmpF inside hexagonal lattice organizations. Figure taken, with permission, from Schabert *et al* (*43*). The scale bar shows a distance of 10 nm. **E**, Distribution of centroid nearest neighbors from AFM data, figures taken with permission from Benn *et. al* (*28*) (*left*) and our CG simulations (*right*) of a subsection of the SOI (located at the bottom right-hand side of main Figure 5 panel) with LPS and PLs removed from in between the OMPs (*pink*), with LPS replaced by PLs in the outer leaflet (*orange*) and the whole SOI (*green*). The greater distribution of distances from the AFM data reflects the larger dataset from the surface of *E. coli* compared to the simulated system. The histogram peak maxima of near-neighbor distances from both data sets are nevertheless similar; ~ 9 nm from AFM data, compared to 9.5 nm from the simulations.

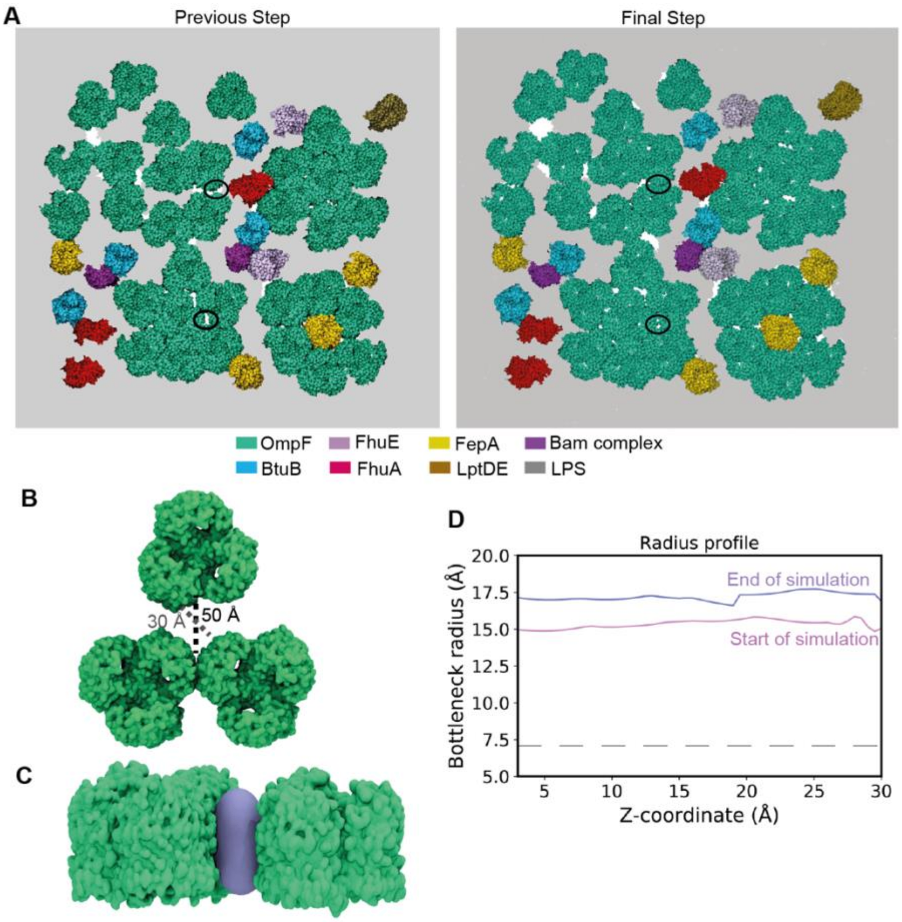

Fig. S18. Simulated removal of LPS from the OM creates holes big enough for the diffusion of vancomycin. A, Snapshot of a heterologous OMP cluster (starting cluster comes from bottom right-hand corner of Fig. 5D) at the middle and end of a simulation whereby LPS was removed from the OM. Lipids (LPS, *grey*) were removed in a step-by-step manner from a 100 x 100 nm patch of clustered OMP. In every step, the system energy was minimized, and the structures were relaxed during an equilibration step. Following several iterations some of the empty membrane regions (*white space*) that resulted from removal of the lipid never close. **B,** A zoom-in of empty membrane region in **A,** identified with black circle, showing the approximate pore size. Distances vary from 30-50 Å depending on where the measurements are taken across the asymmetric hole. **C,** Snapshot of the same conformation from B, with the hole radius depicted in a surface

representation and vancomycin (blue capsule) docked within the hole. **D,** Plot showing the average bottleneck radius of empty membrane regions from the start of the simulation (*purple*) until the end of the simulation (*pink*). Over the duration of the simulation the average radius increases from ~15.4 Å (31 Å diameter) to 17.2 Å (34 Å diameter), remaining well above the R_g of vancomycin (dashed line, 7 Å).

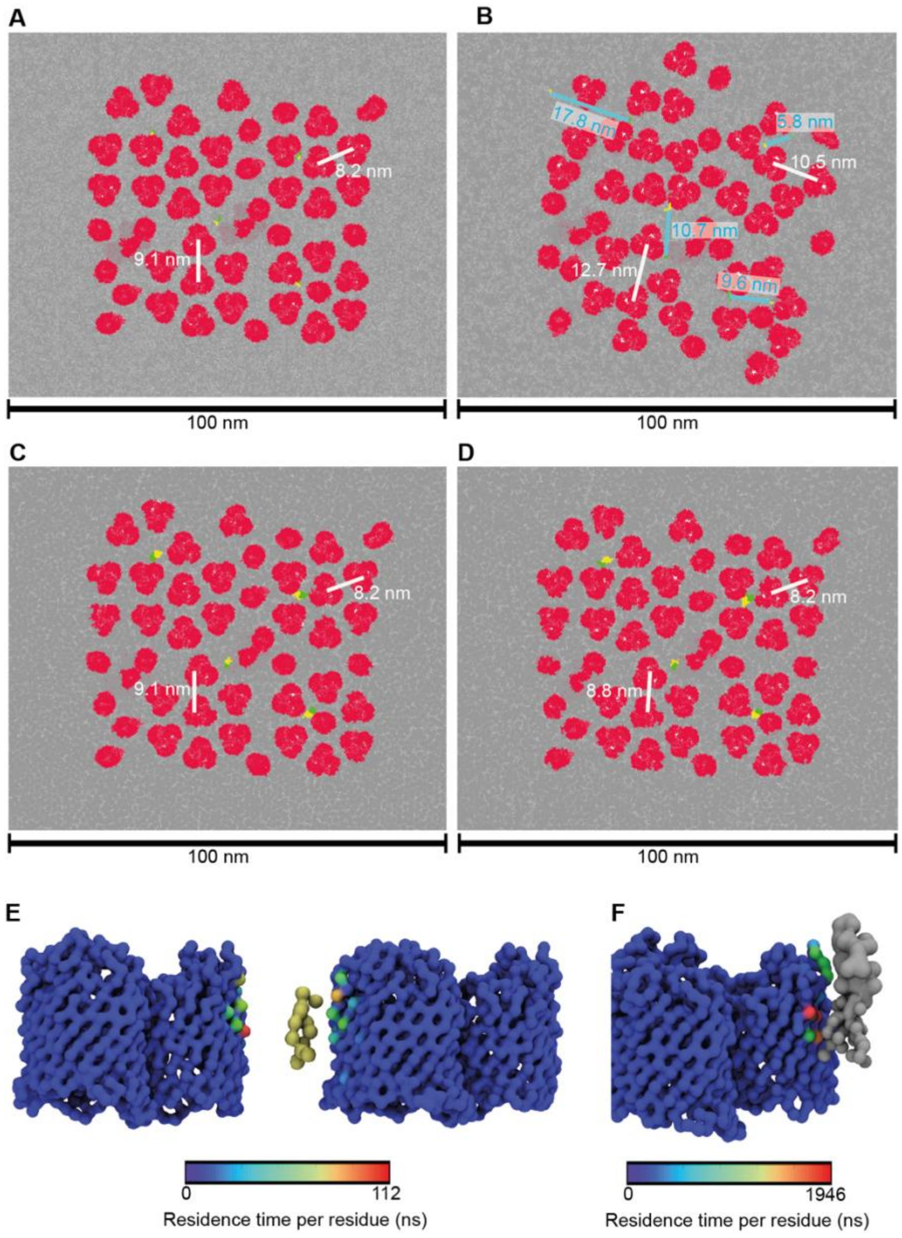

Fig. S19. Increased OMP mobility within a symmetrical phospholipid bilayer destroys the stable, immobile lattice structure of OMP clusters observed in an asymmetric bilayer. A, Snapshot of an OMP cluster (cluster highlighted bottom right-hand corner of Fig. 5D) at the start of the simulation where OMPs (*red*) are embedded within a symmetric PL (*grey*) bilayer. Distances between the center of neighboring OMPs are shown in two cases as 8.2 and 9.1 nm. PL to be measured are identified (*green and yellow*). **B,** Snapshot of OMP island at the end of simulation

colored as in A. Distances between PL identified in A are measured showing movement of 5-18 nm throughout the duration of the simulation. Likewise, the distances between OMPs increases between the start (0 ns) and end of the simulation (2000 ns). **C,** Snapshot of OMP cluster (as in **A**) at the start of a simulation with OMPs (*red*) embedded within an asymmetric LPS/PL (*grey*) membrane. Distances between the center of neighboring OMPs is shown in two cases as 8.9 and 9.1 nm. LPS molecules to be measured are identified (*green and yellow*). **D,** Snapshot of OMP cluster at the end of the simulation colored as in A. LPS molecules identified in A have not moved during the timescale of the simulation (2000 ns), OMPs also display minimal migration (*white line*). Images A-D represent a 100 x 100 nm box. **E,** POPE (*gold*) location is represented between two OmpF trimers. Residues of OmpF are colored according to residence time with POPE molecule displayed. The low residence times suggest a transient interaction between OmpF and PL. **F,** a single LPS molecule (*grey*) is represented relative to OmpF, with OmpF residues colored according to residence time. Residence times increase when in close proximity to LPS and interacting residues are located upper to mid-barrel consistent with experimental data. Residence times of LPS are almost 20 x longer than that of POPE in **E**.

Table S1: Colicin sensitivity of OMP-BPA mutants

Protein	Site	Colicin sensitive?
**OmpF**	A159	No
	F166	No
	V177	Yes
	V196	Yes
	G198	Yes
	Y202	Yes
	G211	Yes
	E234	Yes
	T238	Yes
	Y243	Yes
	A251	Yes
	Q277	Yes
	L281	Yes
	Y285	Yes
	I295	Yes
	D312	Yes
	A312	No
	V333	No
	V356	No
**BtuB**	W164	Yes
	G242	Yes
	R246	Yes
	V335	Yes
	W391	No
	G455	Yes
	V523	Yes
	L569	Yes
**FepA**	A181	Yes
	I255	Yes
	Y331	Yes
	L434	Yes
	I484	Yes
	D553	Yes
	L588	Yes
	V679	Yes

Table S2. Masses of species observed in the BtuB spectra shown in Figures S7-S8

Protein sample	Theoretical mass (Da)	Experimental mass (Da)
BtuB	66,325	66,307
BtuB W164BPA	66,372	66,376
BtuB G242BPA	66,501	66,504/66,459
BtuB L569BPA	66,445	66,448
BtuB G242BPA/G455BPA	66,677	66,698
**Adducts**		
Phospholipid PE	715	
Phospholipid PG	749	
LPS	>3000	
**BtuB-Adducts**		
BtuB G242BPA-LPS	66,501 +>3000	70,220
BtuB G242BPA-PL	66,501+ 715/750	67,163
BtuB L569BPA-LPS	66,445 +>3000	70,209
BtuB W164BPA no UV-LPS	66,372 +>3000	70,135/70,217/70,351
BtuB W164BPA-LPS	66,372 +>3000	70,135/70,216
BtuB G242BPA/G455BPA-PL	66,501+ ~700/1400	67,412
BtuB G242BPA/G455BPA-LPS	66,677 +>3000	70,381/70,460

PE33:1 is PL in LPS samples

Table S3: Masses of species observed in the FepA spectra shown in Figures S7, S9

Protein sample	Theoretical mass (Da)	Experimental mass (Da)
FepA	79,771	79,769
FepA I255BPA	79,891	79,766
FepA I484BPA	79,891	79,906
FepA L588BPA	79,919	
FepA V679BPA	79,905	79,919
**Adducts**		
Phospholipid PE	715	
Phospholipid PG	749	
LPS	>3000	
FhuA	78,742	78,739
FhuE	77,411	77,422
OmpF monomer/trimer	37,084/111,252	111,257
OmpC monomer/trimer	38,307/114,921	114,919
Beta-OG detergent	292	
**FepA-Adducts/ co-purified proteins**		
FepA-LPS	79,771+>3000	83,070/83,167
FepA V679BPA-PL	79,905+715/750	80,630
OmpC-OmpC-OmpF	113,698	113,692
OmpF-OmpF-OmpC	112,475	112,477

Table S4: Masses of species observed in the OmpF spectra shown in Figures S7, S10-S12

**Protein sample**	**Theoretical mass (Da)**	**Average Experimental mass (Da)**
OmpF	111,252	111,250
OmpF V177BPA	111,654	111,708
OmpF V196BPA	111,654	111,712
OmpF G198BPA	111,780	111,842
OmpF Y202BPA	111,462	111,523
OmpF E234BPA	111,564	111,626
OmpF T238BPA	111,648	111,703
OmpF Y242BPA	111,642	111,520
OmpF Q277BPA	111,567	111,631
OmpF L281BPA	111,612	111,635
OmpF Y285BPA	111,462	111,521
OmpF D312BPA	111,606	111,665
OmpF V177BPA/G198BPA	112,182	112,298
**Adducts/co-eluting proteins**		
Phospholipid PE	715	
Phospholipid PG	749	
LPS	>3000	
FepA	79,771	79,770
FhuA	78,742	78,702
FhuA-LPS		81,764
Beta-OG detergent	292	
**BPA-adducts**		
OmpF V177BPA-OG	111,654+292	112,000
OmpF V177BPA-PL	111,645+ ~700 increments	111,710, 112,447, 113,181, 113,910
OmpF V177BPA-LPS or OmpF V177BPA-LPS-PL	111,645 + >3000	115008, 115,744, 116,474
OmpF V196BPA-PL	111,654+ ~700 increments	112,438, 113,174, 113,903
OmpF V196BPA-LPS or OmpF V196BPA-LPS-PL	111,645 + >3000	115,015 115,748, 116,476
OmpF G198BPA-PL	111,780+ ~700 increments	112,581, 113,315, 114,044
OmpF G198BPA-LPS or OmpF G198BPA-LPS-PL	111,780+ >3000	115,141 115,873, 116,602, 118,443, 119,657
OmpF Y202BPA-PL	111,462	112,242, 112,952
OmpF E234BPA-OG	111,564	111,892
OmpF T238BPA-LPS or OmpF T238BPA-LPS-PL	111,385 + >3000	115,048 115,780, 116,512
OmpF T238BPA-PL	111,648 + ~700 increments	111,703,112,440, 113,176
OmpF Y242BPA	111,462 + ~700 increments	111,520, 112,246, 112,981
OmpF Q277BPA-LPS	111,567	114,956
OmpF L281BPA-LPS	111,612+ >3000	115,493
OmpF L281BPA-LPS-PL	111,612+ >3000+~700	116,206

OmpF Y285BPA-PL	111,462 + ~700 increments	112,286, 113,002, 113,739
OmpF Y307BPA	111,462	
OmpF D312BPA-OG	111,606+292	111,957
OmpF V177BPA/G198BPA-PL	112,182+ ~700 increments	113,033, 113,757, 114,498, 115,233, 115,949
OmpF V177BPA/G198BPA-LPS	112,182+ >3000	Complex spectra many peaks

Table S5: LC-MS-MS detection of peptides for OMPs of interest in knockout cell lines complemented with plasmid. *E. coli* BZB1107 *ompF ompC* cells and *E. coli* RK5016 cells (a *btuB* mutant cell line) were supplemented with plasmids expressing *ompF* and *btuB*, respectively. Protein expression was induced with 0.15% arabinose and cells grown for 2 hours at 37 ^{o}C. Cells were harvested and lysed by sonication prior to trypsin digestion of samples. LC-MS-MS analysis was followed by peptide identification in MaxQuant software. OMPs detected following peptide-mass fingerprinting of extracted gel bands from preparations of $OmpF^{BPA}$ and $BtuB^{BPA}$ mutants, were searched for in each lysate sample. No BtuB or OmpC was detected in *E. coli* BZB1107 cells expressing *OmpF* (+OmpF), whilst all OMPs of interest were detected in RK5016 cells expressing *BtuB* (+BtuB).

**OMP**	**BZB1107 +OmpF**	**RK5016 +BtuB**
OmpF	7.81×10^{11}	9.73×10^{9}
OmpC	-	9.56×10^{7}
BtuB	-	9.75×10^{11}
FepA	1.23×10^{10}	5.93×10^{10}
FhuA	7.23×10^{9}	1.5×10^{9}
LptD	2.3×10^{9}	3.72×10^{8}
BamA	2.21×10^{8}	2.01×10^{8}
OmpA	3.96×10^{10}	5.0×10^{9}

Movie S1.

1 μs coarse-grained simulation of the SOI viewed from the LPS outer leaflet (*left panel*) and PL inner leaflet (*right panel*). Key: LPS, *silver spheres*; PLs, *golden spheres*; OmpF trimers, *green surface*; all other OMPs for simplicity shown in *yellow surface*. In both views of the SOI, five LPS-PL pairs have been highlighted (*magenta*) to enable visual tracking of these molecules through the simulation. While the positions of LPS molecules remain largely unchanged, PLs diffuse freely in the inner leaflet.
